# The comparison of differentially expressed microRNAs in Bag-1 deficient and wild type MCF-7 breast cancer cells by small RNA sequencing

**DOI:** 10.3906/biy-2109-48

**Published:** 2021-11-14

**Authors:** Pelin ÖZFİLİZ KILBAŞ, Gizem ALKURT, Pinar OBAKAN YERLİKAYA, Ajda ÇOKER GÜRKAN, Gizem DİNLER DOĞANAY, Elif Damla ARISAN

**Affiliations:** 1 Department of Molecular Biology Genetics and Biotechnology, İstanbul Technical University, İstanbul, Turkey; 2 Department of Molecular Biology and Genetics, İstanbul Kültür University, İstanbul, Turkey; 3 Department of Biomedical Engineering, Biruni University, İstanbul, Turkey; 4 Department of Molecular Biology and Genetics, Biruni University, İstanbul, Turkey; 5 Institute of Biotechnology, Gebze Technical University, Kocaeli, Turkey

**Keywords:** Bag-1 knockout, small RNA sequencing, miRNA, hsa-miR-429, MCF-7, breast cancer

## Abstract

The multifunctional BAG-1 (Bcl-2 athanogene-1) protein promotes breast cancer survival through direct or indirect interaction partners. The number of the interacting partners determines its cellular role in different conditions. As well as interaction partner variability, the amount of BAG-1 protein in the cells could cause dramatic alterations. According to previous studies, while the transient silencing of Bag-1 enhanced drug-induced apoptosis, deletion of BAG-1 could induce stemness properties and Akt-mediated actin remodeling in MCF-7 breast cancer cells. Considering the heterogeneity of breast cancer and the variability of BAG-1 -mediated cell response, it has become essential to determine microRNA (miRNA) functions in breast cancer depending on *Bag-1* expression level. This study aims to compare microRNA expression levels in wt and *Bag-1* knockout (KO) MCF-7 breast cancer cells. hsa-miR-429 was selected as a potential miRNA in BAG-1KO MCF-7 cells because of the downregulation both in bioinformatics and validation qRT-PCR assay. According to predicted mRNA targets and functional enrichment analysis the ten hub proteins that are phosphatidylinositol-4,5-biphosphate 3-kinase catalytic subunit alpha (PIK3CA), kinase insert domain receptor (KDR), GRB2 associated binding protein 1 (GAB1), Rac family small GTPase1 (RAC1), vascular endothelial growth factor A (VEGFA), Cbl proto-oncogene (CBL), syndecan 2 (SDC2), phospholipase C gamma 1 (PLCG1), E1A binding protein p300 (EP300), and CRK like proto-oncogene, adaptor protein (CRKL) were identified as targets of hsa-miR-429. The functional enrichment analysis showed that the most significant proteins were enriched in PI3K/Akt, focal adhesion, cytoskeleton regulation, proteoglycans in cancer, and Ras signaling pathways. It was determined that hsa-miR-429 targeted these pathways in Bag-1 deficient conditions and could be used as a potential therapeutic target in future studies.

## 1. Introduction

The multifunctional Bag-1 protein regulates several cellular pathways, including apoptosis, autophagy, proliferation, invasion, and metastasis in cancer cells (Ozfiliz et al., 2015; Kilbas et al., 2019; Kizilboga et al., 2019; Turk et al., 2021). Bag-1 has three different isoforms (Bag-1S, 33 kDa; Bag1M 46 kDa; and Bag-1L 52 kDA) that have distinct anti-apoptotic effects in breast cancer. Bag-1L isoform has a role in the increased cell viability and resistance to apoptosis (Liu et al., 2009). Moreover, according to the isoform-specific interactome analysis, protein processing, and ER-associated protein degradation (ERAD) pathways were controlled by Bag-1 expression in protein homeostasis of breast cancer (Can et al., 2021). It has been well implicated that increased Bag-1 expression is associated with breast cancer progression and invasion (Brimmell et al., 1999). The clinical relevance of Bag-1 was shown as a prognostic biomarker in Oncotype DX and PAM50 assay, which highlighted its role in breast cancer (Paik et al., 2004; Bernard et al., 2009). The immunohistochemical studies showed that Bag-1 cytoplasmic positive staining scores were correlated with breast cancer progression in estrogen receptor-positive tissue samples (Turner et al., 2001). However, downregulation of Bag-1 expression sensitized tamoxifen-resistant cells to tamoxifen and enhanced the apoptotic potential of cisplatin and paclitaxel in MCF-7 cells (Kilbas et al., 2019; S. Lu et al., 2019). Moreover, stable knockout of Bag-1 enhanced the stemness properties of cells through increased expression of stem cell markers in mice and caused stress-mediated hyperactivation of Akt and actin cytoskeleton remodeling in MCF-7 cells (Tang et al., 2017) (Ozfiliz-Kilbas P et al., n.d.). These findings highlighted that Bag-1 expression is critical in cellular homeostasis.

miRNAs are 22 nucleotide length endogenous, single-stranded small noncoding RNAs and negatively regulate gene expression and affect several cellular pathways. miRNAs regulate post-transcriptional mechanisms by degrading mRNA and inhibiting target protein translation through sequence-specific binding to the 3′-untranslated region (UTR) of target mRNA or coding sequences (Ghildiyal & Zamore, 2009; Hausser et al., 2013). miRNAs have essential biological functions in cell proliferation, apoptosis and differentiation, drug resistance, and mostly in cancer (Volinia et al., 2006). The tumor-associated miRNAs are classified as a tumor suppressor (TS)-miRNAs and cancer-causing onco-miRNAs (Takahashi et al., 2013). Dysregulation on the expression profiles of miRNAs have been commonly associated with cancer development, cellular transformation, and tumorigenesis (Melo & Esteller, 2011).

The association between heterogeneous characteristics of breast cancer and differential expression of miRNAs during breast cancer progression made it important to elucidate miRNA functions and the profiling of miRNA-targeted genes in breast cancer. Although numerous studies have shown that different expression levels of BAG-1 show different functions in breast cancer, miRNA profiles of Bag-1-deficient cells associated breast cancer development remains largely unknown. The current study aimed to screen the differential expressed miRNAs and determine the potential targeted genes and pathways in *Bag-1* deficient conditions in MCF-7 breast cancer cells. High-throughput small RNA sequencing, identification of differentially expressed miRNAs, and prediction of target genes and networks in wt and BAG-1KO MCF-7 cells showed that PI3K/Akt signaling, ECM-receptor interaction, proteoglycans in cancer, and TGF-β signaling pathways were highly enriched in BAG-1KO samples compared to wt samples. Experimental qRT-PCR validation of differentially expressed miRNAs confirmed that hsa-miR-429 significantly decreased in BAG-1KO samples. The protein-protein interaction (PPI) network of potential gene targets of hsa-miR-429 showed that PI3K/Akt signaling and cell adhesion pathways are highly enriched in BAG-1KO cells when hsa-miR-429 is downregulated. Alternations in target miRNA expression due to directly or indirectly affected *by Bag-1* deficiency provide novel results for the relationship between different *Bag-1* expressions and miRNA profiles in breast cancer. The altered miRNA-associated potential gene and pathway network, obtained from this study, can be used as preliminary data for future studies.

## 2. Materials and methods

### 2.1. Cell culture of wt and BAG-1KO MCF-7 cells

The wt MCF-7 (HTB-22) human breast cancer cells were maintained in Dulbecco’s Modified Eagle Medium (DMEM, PanBiotech, Germany) augmented with 10% Fetal Bovine Serum (FBS, PanBiotech, Germany), 100-units/ml penicillin, and 100-μg/ml streptomycin (Pan Biotech, Germany). Cells were kept at 37°C in a humidified 5% CO_2_ incubator. BAG-1KO MCF-7 cells were generated through co-transfection of Bag-1 CRISPR/Cas9 Knockout (KO) plasmid (sc-417179) (Santa Cruz Biotechnology, USA) and Bag-1 Homology Directed Repair (HDR) plasmid (sc-417179-HDR) (Santa Cruz Biotechnology, USA) into MCF-7 cells. Control CRISPR/Cas9 Knockout plasmid (sc-418922) was transfected into MCF-7 cells for negative control. Cells were transfected with UltraCruz Transfection Reagent (Santa Cruz Biotechnology, USA) for 48 h according to the manufacturer’s protocol. Following transfection cells were treated with puromycin (Santa Cruz Biotechnology, USA) for 48h and selected by single-cell dilution process. Comparison of wt and Control KO cells showed similar survival, proliferation and growth potentials. After validation and characterization of Bag-1 deficiency in MCF-7 cells, one colony showing the lack of Bag-1 expression was selected for further experiments (Ozfiliz-Kilbas P et al., n.d.). The confirmation of knockout of Bag-1 in MCF-7 cells were shown in Figure S1. BAG-1KO MCF-7 cells were also grown in the same conditions with wt MCF-7 cells.

### 2.2. RNA extraction and quality control

According to the manufacturer’s protocol, the total RNA of wt and BAG-1KO MCF-7 cells were isolated using SPLIT RNA Extraction Kit (Lexogen, Austria). According to the manufacturer’s instructions, total RNA quality, concentration, and integrity were measured with the Agilent 2100 Bioanalyzer (Agilent Technologies, USA).

### 2.3. Construction of sequencing libraries

The total RNA from two biological replicates for each group was used in sequencing experiments. Small RNA libraries were constructed directly from total RNA using the Small RNA-Seq Library Prep Kit for Illumina Platforms (Lexogen, Austria) following the manufacturer’s instructions. Small RNAs are ligated at 3′, and 5′ ends, respectively, and converted into cDNA.

### 2.4. RNA-seq and data analysis of small RNA sequencing

High-throughput RNA-seq was performed by Illumina MiSeq Kit V3 sequencing platform (Illumina, USA). The data were uploaded to the Galaxy web platform, and all downstream analyses were done at usegalaxy.org (Afgan et al., 2018). FastQC reported the quality check of raw reads with sequence quality scores, sequence-based quality scores, sequence GC and Kmer content, duplicate sequences, overrepresented sequences and overall sequence length distribution read distribution plots. The FastQC quality control reports from two biological replicates of wt and BAG-1KO MCF-7 cells were shown in Figure S2 A–D. The Illumina small RNA-Seq Library kit adapter sequence 5′-TGGAATTCTCGGGTGCCAAGGAACTCCAGTCAC-3′ was trimmed, leaving a minimum overlap length=5 and maximum error rate= 0.1 by Cutadapt. The minimum length was selected 18 to discard sequences fewer than 18 base pairs in size, and the total length was selected 35 to exclude more than 35 base pairs in length. The quality of trimmed reads was controlled by FastQC and shown in Figure S3 A–D. The trimmed reads were aligned to the reference human genome GRCh37-hg19 by Bowtie2 with BAM format output. The aligned reads were annotated to mature human miRNA (http://www.mirbase.org/ftp.shtml) by featureCounts and were shown in Figure S4. The144 miRNAs were found in either in two replicated wt or BAG-1KO samples (Figure S5), 88 miRNAs were only present in two replicated wt samples (Figure S6), and 71 miRNAs were only present in two replicated BAG-1KO samples with at least 1 TPM (transcripts per million) (Figure S7). The overall alterations between wt and BAG-1KO MCF-7 cells were analyzed by Rosalind OnRamp software (Figure S8 A–B).

### 2.5. Identification of differentially expressed miRNAs in wt and BAG-1KO cells

To analyze differential expressions of 2576 miRNA counts obtained from featureCounts annotation, DESeq2 differential expression analysis was performed. The output of DESeq2 gives the fold changes (log2) of genes based on the factors (untreated/treated) and generates a tabular file. In our study, the fold changes of “Bag-1KO samples” against “wt” samples were compared, and the result was shown in Figure S9. The false discovery rates (FDR) ≤0.01 was accepted significantly. The fold change determined the expression ratio between wt and BAG-1KO cells.

### 2.6. The functional analysis of miRNAs using DIANA-miRPath v3.0 prediction algorithm

DIANA-miRPath v.3.0 web platform was used to investigate the molecular function of miRNAs and enrichment of pathways. The algorithm performs a statistical model mentioned in Vlachos et al. (Vlachos et al., 2015) (http://snf-515788.vm.okeanos.grnet.gr/). Functional enrichment of target genes for downregulated and upregulated miRNAs was also performed on DIANA-miRPath v.3.0. KEGG pathway analysis was performed to predict target genes and molecular pathways associated with differentially expressed miRNAs in BAG-1KO and wt samples. GO analysis was performed to annotate the biological processes, cellular components, and molecular functions of miRNA-associated mRNA targets. Both functional enrichments analyzed FDR p-value <0.05 were enriched using Fisher’s exact t-test hypergeometric distribution.

### 2.7. Validation of selected differentially expressed miRNAs by quantitative polymerase chain reaction (RT-qPCR)

Total RNA, including smaller than ~200 nucleotides was extracted from MCF-7 wt and BAG-1KO cells using miRNeasy Mini Kit (Qiagen, USA), and the reverse transcription of total RNA containing miRNA was performed using miScript II RT Kit (Qiagen, USA) according to the manufacturer’s protocol. The qRT-PCR experiment was performed using miScript SYBR Green PCR Kit (Qiagen, USA), and the cycling conditions were as follows: initial activation 95 °C for 15 min, followed by 40 cycles of denaturation 94 °C for 15 s, annealing 55 °C for 30 s, extension 70 °C for 30 s. The reactions were performed in two replicates and the selected miRNA primer sequences were listed in Table 1. RNU6 small nuclear RNA was used as a standard internal control and to normalize expression profiles. Relative expression of miRNA samples was calculated using the 2^−ΔΔCq^ methods. Data analyses were performed via GraphPad Prism 8.0.0.

### 2.8. Protein-Protein Interaction (PPI) Network Analysis

Search Tool for the Retrieval of Interacting Genes/Proteins (STRING) database was used to predict protein-protein interactions derived from genomic context, high-throughput lab experiments, co-expression, text mining database knowledge (Szklarczyk et al., 2019). The active interaction sources were selected from experiments and databases for reliable results. Moreover, the minimum required interaction score was set at high confidence (0.700). The mRNA regulatory network was visualized by Cytoscape open-source platform version 3. 8. 2 (Shannon et al., 2003).

### 2.9. Statistical analysis

The differential expression of miRNAs was performed using the Galaxy web platform, and the FDR ≤0.01 expression results were considered as significant. Then, the functional KEGG and GO enrichment analysis were performed using FDR <0.05. The qRT-PCR results were calculated using 2^−ΔΔCq^ and RNU6 was used as a normalization control. For the analyses of results “Two-way ANOVA followed by Sidak’s multiple comparisons tests was performed using GraphPad Prism 8.0.0, Graphpad Software, San Diego, California USA, www.graphpad.com”. To predict protein-protein interaction STRING was used at 0.7 high confidence ratio, and interaction network was visualized by Cytoscape web-platform using CytoHubba application in the Cytoscape database using the ˚the degree˚ calculation method. The Database for Annotation, Visualization and Integrated Discovery (DAVID) bioinformatics tools (https://david.ncifcrf.gov/) was performed for the functional characterization of mRNA targets.

## 3. Results

### 3.1. Small RNA sequencing and determination of differentially expressed miRNAs in wt and BAG-1KO cells

**Figure 1:**
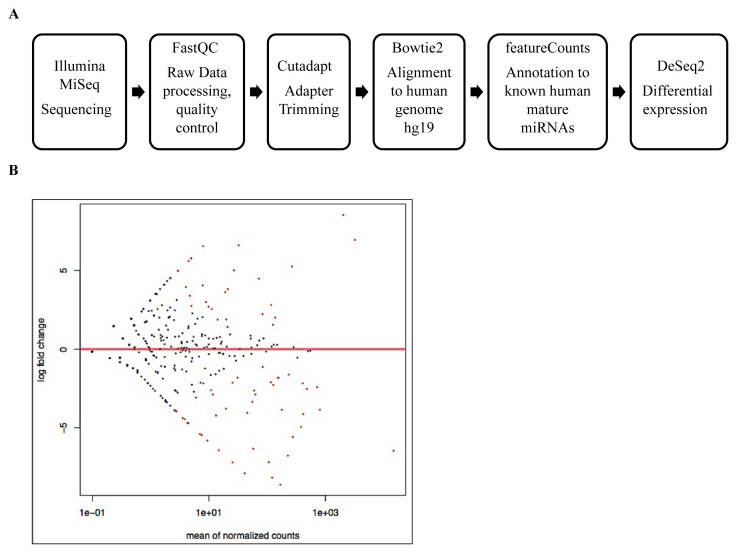
A) The pipeline of small RNA sequencing of wt and BAG-1KO MCF-7 samples. The scheme illustrated the five steps of RNA sequencing analysis: the quality control of raw reads, the adapter trimming, alignment, annotation, and differential expression of miRNAs in BAG-1KO samples compared to wt cells. B) MA plot of differentially expressed miRNAs between BAG-1KO and wt MCF-7 samples. Y-axis showed the log2 fold change, and the x-axis showed the average expression. Red dots represented the differentially expressed miRNAs, and gray dots were shown in no change. Negative values of fold changes represented downregulated, positive values represented upregulated miRNAs.

High throughput next-generation sequencing was performed from two biological replicates of wt and BAG-1KO MCF-7 cells using the computational workflow in Figure 1A. Following sequencing, the quality of raw reads was controlled by FastQC. According to the basic quality report statistical report, the total number of raw sequences in wt and BAG-1KO samples was processed and the adapter sequence was removed by the Cutadapt tool. The trimmed reads with less than 18 bp were excluded for reliable analysis. The trimmed and processed reads of wt and BAG-1KO bases were sequentially aligned to the GRCh37-hg19 human genome using Bowtie2. The sequence counts for 2576 miRNAs of two biological replicates of wt and BAG-1KO samples annotated in the hg19 mature human miRNAs using the featureCounts tool. The differential expression analyses of 2576 miRNA counts were performed by DESeq2 and the mean average (MA) plot of differentially expressed miRNAs between BAG-1KO and wt MCF-7 samples were shown in Figure 1B. The Following the Benjamini-Hochberg correction method, False Discovery Rates (FDR) ≤0.01 was set to obtain significantly differentially expressed miRNAs, and with this correction method, 25 known miRNAs were found significantly differentially expressed in BAG-1KO reads comparing wt reads. According to the comparison results in BAG-1KO and wt MCF-7 cells, while 14 different miRNA targets were downregulated in BAG-1KO cells, 11 of the significantly upregulated miRNA targets were determined (Table 2).

### 3.2. Determination of predicted target genes of differentially expressed miRNAs in wt and BAG-1KO samples

To determine the potential functions of differentially expressed miRNAs and further explore miRNAs-associated target genes controlling molecular pathways, in silico DIANA-miRPath v3.0 miRNA target prediction algorithm was performed incorporating KEGG and GO functional enrichment analysis.

**Figure 2:**
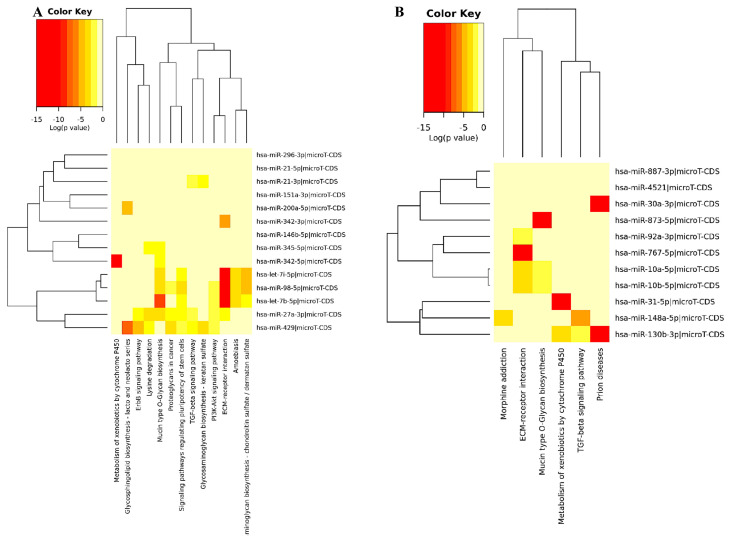

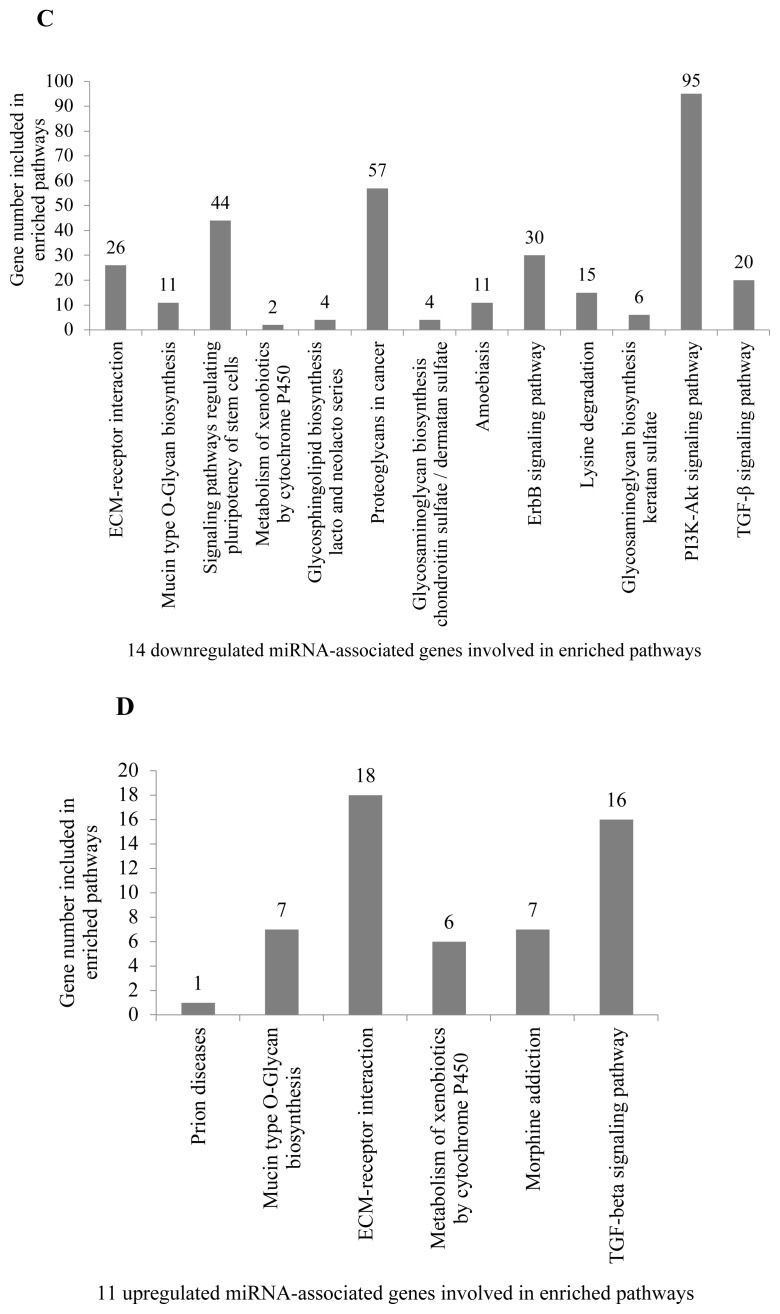
Pathway enrichment KEGG analysis results were extracted from DIANA-miRPath v.3.0 for downregulated 14 miRNA (A) and upregulated 11 miRNA (B) targets in BAG-1KO MCF-7 samples compared to wt MCF-7 samples based on the microT-CDS (v.5.0) miRNA input. Red colors represented the higher level of pathway enrichment of the miRNA target genes. Functional KEGG enrichment was analyzed using threshold 0.7 and FDR p-value <0.05. C) The bar graph showed the downregulated mRNA target gene numbers involved in enriched pathways obtained from KEGG analysis. D) The bar diagram showed the downregulated mRNA target gene numbers involved in enriched pathways obtained from KEGG analysis.

KEGG pathway enrichment analysis: We first explored the potential mRNA targets and significantly enriched pathways of all downregulated 14 miRNAs in Bag-1KO cells compared with wt MCF-7 cells. According to KEGG pathway analysis, we found that signaling pathways regulating pluripotency, PI3K/Akt signaling mechanism, Mucin type O-Glycan biosynthesis, ECM-receptor interaction, proteoglycans in cancer, and TGF-β signaling pathway were targeted in Bag-1KO MCF-7 cells (Figure 2A). The upregulated 11 miRNAs were enriched in pathways involving morphine addiction, ECM-receptor interaction, mucin-type O-Glycan biosynthesis, and metabolism of xenobiotics by cytochrome p450, TGF-β signaling pathway, and prion diseases (Figure 2B). Downregulated miRNA-associated 95 target genes as the highest number were involved in PI3K/Akt signaling pathway. Then, proteoglycans in cancer were found as the second enriched pathway with 57 target gene involvement. 44 genes were involved in the pluripotency of stem cell signaling pathways in Bag-1KO MCF-7 samples (Figure 2C). However, it was observed that the number of enriched pathways of the upregulated miRNA-targeted genes was a small number compared to the downregulated miRNA-targeted genes enriched pathways. ECM-receptor interaction and TGF-β signaling pathways were the highly enriched pathways with the 18 and 16 genes associated with upregulated miRNAs (Figure 2D). The enriched KEGG pathways, differentially expressed miRNAs, and miRNA-associated target genes of 14 downregulated and 11 upregulated miRNAs were shown in Table 3 and Table 4, respectively.

**Figure 3:**
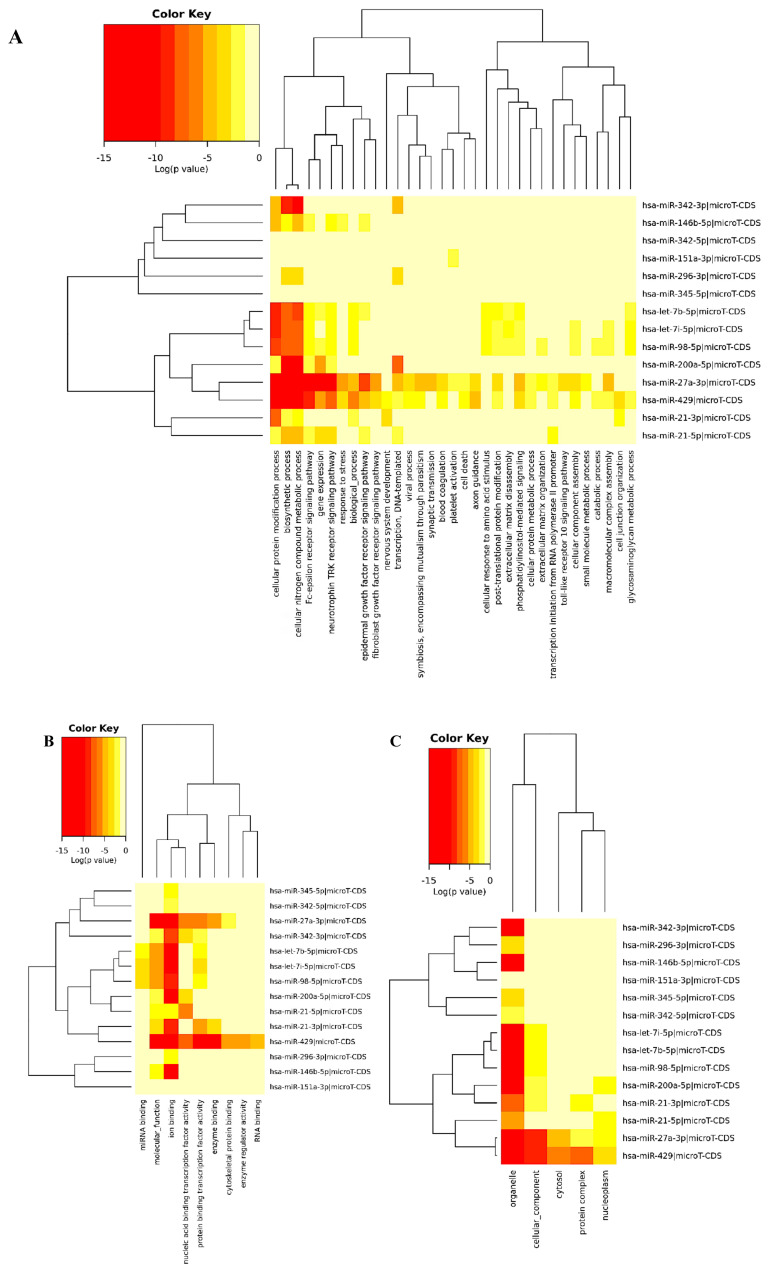

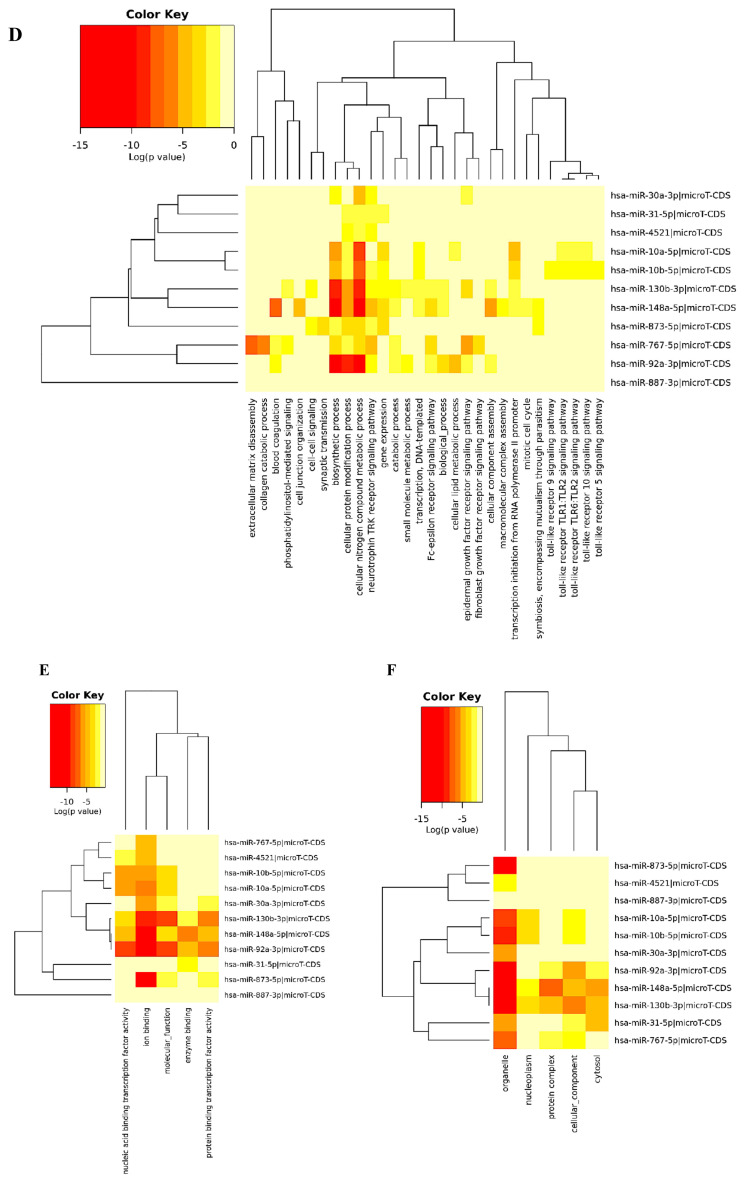
GO enrichment analysis of 14 downregulated miRNAs at three levels: biological process (A), molecular functions (B), and cellular component (C). GO enrichment analysis of 11 upregulated miRNAs at three levels: biological process (D), molecular functions (E), and cellular component (F). The darker colors represented a higher degree of enrichment. FDR p-value <0.05 was considered as significant in functional enrichment analysis.

GO functional annotation analysis: The significance of downregulated and upregulated miRNAs was analyzed using GO analysis in DIANA-miRPath v.3.0 by examining biological processes, molecular functions, and cellular components. The downregulated 14 miRNAs were enriched in biological processes involving gene expression, Fc-epsilon receptor signaling pathway, neurotrophin TRK receptor signaling pathway, cellular protein modification process, biosynthetic process, epidermal growth factor receptor signaling pathway, response to stress, cytoskeletal protein binding, extracellular matrix disassembly and organization and cell-cell signaling (Figure 3A). The molecular functions of downregulated miRNAs were enriched in nucleic acid and protein binding transcription factor activity, ion binding, enzyme binding, cytoskeletal protein binding, enzyme regulator activity, RNA, and mRNA binding (Figure 3B). The enriched cellular components include organelle, protein complex, nucleoplasm, and cytosol (Figure 3C). The detailed GO enrichment analysis, including p-value and gene number of 14 downregulated miRNAs, was shown in Figure S10 A–C.

The biological process of upregulated 11 miRNAs was enriched in the biosynthetic process, cellular protein modification process, cellular nitrogen compound metabolic process, gene expression, phosphatidylinositol-mediated signaling, mitotic cell cycle, and cell junction organization (Figure 3D). The molecular function category of GO terms showed nucleic acid binding transcription factor activity, ion binding, protein binding transcription factor activity, and enzyme binding pathways of 11 upregulated miRNAs (Figure 3E). The enriched cellular components include organelle, cytosol, protein complex, and nucleoplasm (Figure 3F). The detailed GO enrichment analysis, including p-value and gene number of 11 upregulated miRNAs, was shown in Figure S11A–S11C.

### 3.3. Experimental qRT-PCR validation of 14 key miRNAs

**Figure 4:**
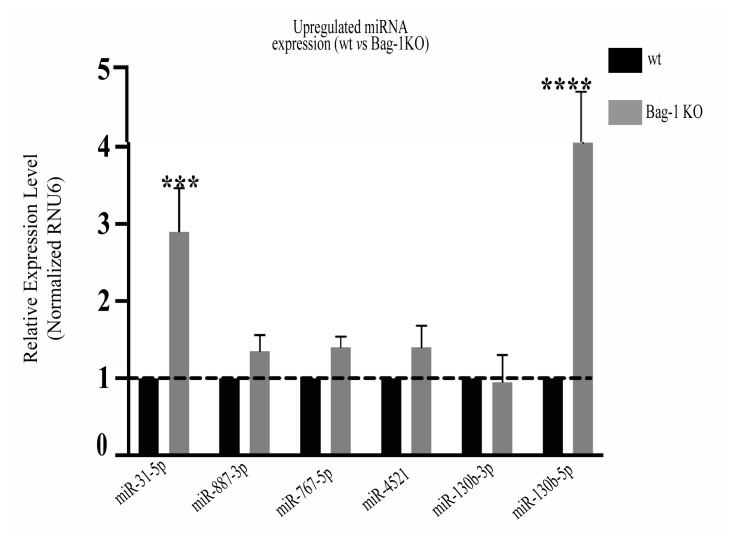
qRT-PCR validation of differentially expressed selected miRNAs in wt and BAG-1KO samples. The expression patterns of significantly changed selected 14 miRNAs were validated by qRT-PCR analysis and calculated using the 2^−ΔΔCq^ methods. a) The bar diagram represented the downregulated expression of selected miRNAs in BAG-1KO MCF-7 samples compared to wt samples. b) The bar graph showed the upregulated expression of selected miRNAs in BAG-1KO MCF-7 samples compared to wt samples. RNU6 was used as a reference gene for qRT-PCR normalization. All data collected from three independent experiments and the designed primer sequences were shown in Table 1 (***p = 0.0002, ****p < 0.0001).

The predicted expression profiles of significantly differentially expressed 14 miRNAs of 25 known miRNAs were validated using qRT-PCR analysis. The relative expression profiles of hsa-miR-27a-3p, hsa-miR-151a-3p, and hsa-miR-342-3p showed ~10-fold; hsa-miR-429, and hsa-miR-146b-5p showed ~4-fold; hsa-miR-21-5p, hsa-miR-200a-5p, and hsa-miR-345-5p showed ~2.5-fold downregulation in Bag-1KO samples compared to wt samples. Besides, hsa-miR-31-5p showed 2.9-fold, and hsa-miR-10a-5p showed 4-fold upregulation in Bag-1KO cells compared to wt cells (Figure 4).

### 3.4. Selection of hsa-miR-429 as a potential target and analyzing the PPI Network of genes targeted by hsa-miR-429

According to the results of differentially expressed 25 miRNAs in Bag-1KO samples, hsa-miR-429 was found the third most downregulated expression profile of the 14-downregulated miRNAs after hsa-miR-21-3p and hsa-miR-21-5p (Table 2). It was also involved in 8 KEGG terms out of 13 enriched KEGG pathways of downregulated miRNA-associated target genes (Table 3), and experimentally validated with the confirmation of its downregulated expression profile (Figure 4). The results of hsa-miR-429 were found to be consistent in both sequencing and experimental PCR analysis, as they showed a similar downregulation profile. Therefore, hsa-miR-429 was selected as a potential target for Bag-1KO samples in further analyses.

The target genes of hsa-miR-429 from DIANA-miRPath v.3.0 were compared in the miRDB online database used for miRNA target prediction (http://mirdb.org/cgi-bin/search.cgi). Potential gene targets of hsa-miR-429 were identified from common predicted target genes in both DIANA-miRPath v.3.0 (Table 5).

**Figure 5:**
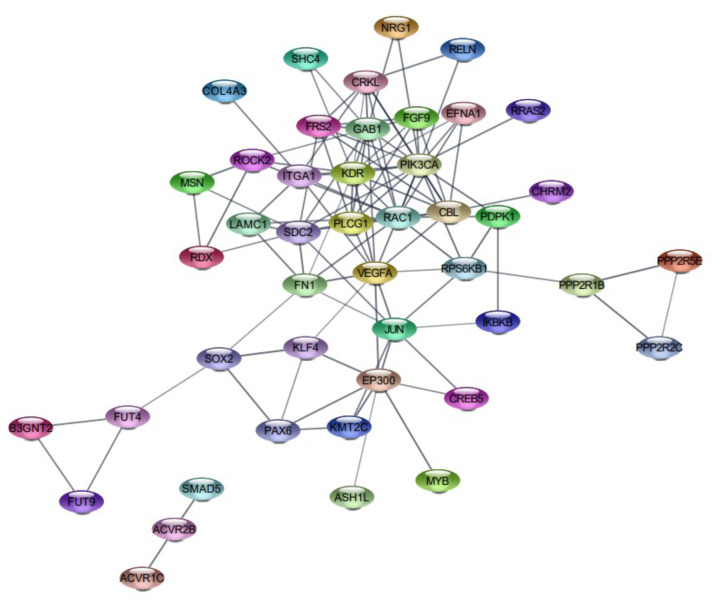
PPI network of predicted targets for hsa-miR-429 using Cytoscape visualization database. The figure illustrates the putative interaction of genes targets of hsa-miR-429 and consisted of 57 nodes and 97 edges.

**Figure 6:**
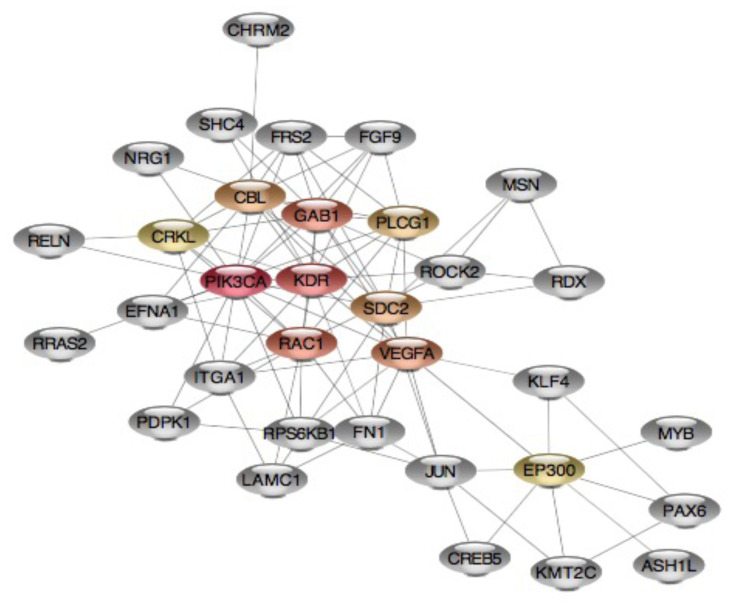
PPI-network for the critical targets of hsa-miR-429. Circular yellow nodes represented the top 10 hub genes of predicted target genes for hsa-miR-429, grey nodes represented the significant targets of hsa-miR-429.

The PPI network of the predicted gene targets for downregulated hsa-miR-429 was developed using the STRING database, and the visualization of the PPI network was established through Cytoscape software. The network was composed of 57 nodes and 97 edges (Figure 5). The top ten hub proteins for the target of hsa-miR-429, which are the primary nodes of several interaction partners, were identified as phosphatidylinositol-4,5-biphosphate 3-kinase catalytic subunit alpha (PIK3CA), kinase insert domain receptor (KDR), GRB2 associated binding protein 1 (GAB1), Rac family small GTPase1 (RAC1), vascular endothelial growth factor A (VEGFA), Cbl proto-oncogene (CBL), syndecan 2 (SDC2), phospholipase C gamma 1 (PLCG1), E1A binding protein p300 (EP300), and CRK like proto-oncogene, adaptor protein (CRKL). The PPI network of the top ten hub genes was shown in Figure 6 and Table 6.

### 3.5. Functional annotation and pathway enrichment analyses for the target gene of hsa-miR-429

The GO functional annotation and KEGG pathway enrichment analyses of the top ten hub genes included essential proteins of predicted target genes for hsa-miR-429, were performed using the DAVID database. The most significant GO terms were listed in Table 7, including positive regulation on gene expression, phosphatidylinositol-mediated signaling, angiogenesis, cell migration, and cell adhesion in the biological process category. Moreover, plasma membrane, cytoplasm, intracellular membrane, and focal adhesion were found in the cellular compartment category, protein binding, phosphatidylinositol-4,5-biphosphate3-kinase activity, protein kinase, receptor tyrosine kinase, and integrin binding were observed in the molecular function category. The KEGG pathway enrichment analysis revealed that the most significant genes targeted by hsa-miR-429 were enriched in proteoglycans in cancer, PI3K-Akt signaling, focal adhesion, cancer pathways, actin cytoskeleton regulation pathway, and Ras signaling pathway, which was listed in Table 8.

## 4. Discussion

BAG-1 protein is one of the critical targets in breast cancer cells, which functions as a co-chaperone for HSP70/HSC70 proteins, triggers the cellular survival and results in a high proliferation rate compared to normal cells (Song et al., 2001; Ozfiliz et al., 2015). Elevated protein levels of BAG-1 determined in metastatic cancer patients and associated with decreased drug-induced apoptosis in breast cancer (Kizilboga et al., 2019). On the contrary, elimination of BAG-1 expression through silencing increased the efficiency of therapeutics (Kilbas et al., 2019). Following this, BAG-1 expression levels were shown as a limiting factor in the efficiency of breast cancer therapies.

miRNAs, the posttranscriptional regulators, are 18–23 nucleotide long noncoding RNAs (Castro, Moreira, Gouveia, Pozza, & Mello, 2017). They have regulatory roles controlling the development, progression, and metastasis of breast cancer at the posttranscriptional phase; however, these miRNAs’ functions and expression profile differs according to the subtype of breast cancer (Kurozumi et al., 2017). Different miRNAs function independently to repress a single target, or combinatorial cause more dramatic effects in repressing a target (Linsen, Tops, & Cuppen, 2008). Although previous studies have demonstrated miRNA-mediated breast cancer development, the miRNA expression profile in Bag-1 deficient-breast cancer cells is unknown (Singh & Mo, 2013). One of the previous studies showed that the tumor suppressor hsa-miR-138 expression was decreased in the tumor tissues, and the regain of miR-138 expression enhanced apoptosis of gallbladder cancer through directly targeting the 3′-UTR sites of *Bag-1*. The loss and gain-of-function studies confirmed that the Bag-1 is a direct target for miR-138 (Ma et al., 2015). Moreover, miR-28-5p was found as a tumor suppressor directly targeting and deregulating *Bag-1* in B cells (Schneider et al., 2014).

Here, we discuss the differential expression profiles of miRNAs in Bag-1 deficiency leading to breast cancer progression in MCF-7 cells. Following small RNA sequencing analyses, 25 significant differentially expressed miRNAs were observed, 14 miRNAs were found downregulated, and 11 miRNAs were upregulated in BAG-1KO samples compared to wt MCF-7 samples. It was noteworthy that the enriched pathways associated with upregulated miRNAs were less in number compared with downregulated miRNAs enriched pathways. To eliminate the false-positive rates of prediction algorithms, the relative expression of 14 miRNAs selected out of 25 differentially expressed miRNAs to sample different log2 fold change number was experimentally validated by qRT-PCR analyses.[Table t7-turkjbiol-46-2-118]

According to the results of downregulated miRNA profiles in BAG-1KO samples, previous studies showed that hsa-miR-21 is required for cell proliferation, PI3K/Akt activation, and epithelial-mesenchymal transition (EMT) in MDA-MB-231 and SKM-1 cells (Li et al., 2018; Arisan et al., 2021). In addition, hsa-miR-27a-3p has been identified as an onco-miR and promotes proliferation and migration of colorectal and triple negative breast cancer cells through targeting BTG-1 and GSK3β, respectively (C. Su, Huang, Liu, Liu, & Cao, 2019). hsa-miR-342 and hsa-miR-146 are associated with estrogen levels, and have a role in negative regulation of BRCA1 in breast cancer (AI et al., 2011; Crippa et al., 2014). hsa-miR-345 has been known with its inhibitory role on tumor metastasis and induction of apoptosis in hepatocellular and pancreatic carcinoma (Bao et al., 2014; M et al., 2017). hsa-miR-151 was found to repress migration in breast cancer through targeting TWIST1 (Yeh et al., 2016). In addition, hsa-miR-98-5p has been known for its anti-oncogenic function in breast cancer (Shi, Wang, Wang, & Gu, 2019). The miR-200 family is one of the most studied miRNAs associated with EMT and metastasis. Significant downregulation of the miR-200 family, which consists of miR-141, miR-200a, miR-200b, miR-200c, and miR-429, regulates the expressions of mesenchymal markers ZEB1, ZEB2, TWIST1, TWIST2, Snai1, and Snai2 and promotes EMT in breast cancer (Bockmeyer et al., 2011). Previous studies demonstrated that miR-200a showed decreased expression profile in EMT-induced cancer cells, but overexpression was associated with downregulated N-cadherin, ZEB1, vimentin, and upregulated E-cadherin profile (Y. Lu et al., 2014; Pichler et al., 2014). miR-429, a member of the miR-200 family, has been reported by its inhibitory role on EMT, tumor migration and metastasis in hepatocellular, nasopharyngeal and breast cancer (Guo, Zhao, Zhang, Liu, & Sun, 2018). Loss of function studies showed a decreased expression profile in breast cancer, inducing EMT and promoting bone metastasis directly targeting ZEB1 transcription factor (Ye et al., 2015).

In the current study, hsa-miR-429 was consistently downregulated in BAG-1KO MCF-7 cells in both bioinformatics and qRT-PCR validation assays. Moreover, hsa-miR-429 was involved in downregulated miRNAs-associated KEGG enrichments in 8 of a total 13 pathways; therefore, we suggested that hsa-miR-429 and its gene targets can be a potential purpose to explain the survival properties of BAG-1KO cells. According to microRNA targets database (miRDB) and Targetscan, has-miR-429 significantly binds to 3′UTRs of *Bag-5* and *Bag-6*, which are also member of *Bag-1* gene family, which possess antiapoptotic function in the cells due to presence of Bag domain to regulate Hsp70 co-chaperone activity. Although hsa-miR-429 is not directly bound to *Bag-1* mRNA to regulate its function, it was observed from our results that the predicted gene targets of hsa-miR-429 were PIK3CA, KDR, GAB1, RAC1, VEGFA, CBL, SDC2, PLCG1, EP300, and CRKL, which are significantly enriched in PI3K/Akt signaling, cell migration, cell adhesion, focal adhesion, integrin binding, and actin cytoskeleton regulation.

Although there is no direct evidence that miR-429 targets PIK3CA, previous studies showed that it inhibits cell proliferation and migration through targeting AKT serine/threonine kinase 1 (AKT1) involved in PI3K/Akt pathway in renal cell carcinoma (Z. Su et al., 2020). Similarly, although direct interaction of KDR with hsa-miR-429 has not been demonstrated, it was observed that hsa-miR-429 suppresses breast cancer invasion by inhibiting the Wnt/β-catenin pathway through potentially targeting SOX2, CBL, FN1, KDR, KLF4, NR3C1, QK1, RBFOX3, SYNJ1, and VEGFA detected by CytoHubba algorithm in Cytoscape (Zhang et al., 2020). GAB1 was found to be directly targeted by hsa-miR-200a, a miRNA family including hsa-miR-429, to suppress cell invasion and migration of hepatocellular carcinoma (Wang & Sun, 2017). hsa-miR-429 decreased the expression of VEGFA in human endothelial cells by directly inhibiting Hypoxia-inducible factor 1 (HIF1) (Bartoszewska et al., 2015). In breast cancer, it was determined that hsa-miR-200bc/429 cluster directly targets PLCG1 and regulates EGF-driven invasion. (Uhlmann et al., 2010) Moreover, hsa-miR-429 also directly targets and negatively regulates (v-crk sarcoma virus CT10 oncogene homolog (avian)-like) CRKL adaptor protein in breast cancer to promote cell migration and invasion (Guo et al., 2018). Although several studies determined the direct or indirect network between PIK3CA, KDR, GAB1, VEGFA, PLCG, and CRKL and hsa-miR-429 in various cancers, the functional regulation of these genes in *Bag-1* deficiency conditions is still needed to elucidate. However, there is no direct or indirect interaction network between hsa-miR-429 and RAC1, CBL, SDC2 and EP300. Therefore, these preliminary screening data can be used to investigate the regulation of survival and proliferation conditions of Bag-1KO breast cancer cells.

## Supplementary Materials

Figure S1 Determination of the loss of Bag-1 expression by immunoblotting. GAPDH was used as a loading control.

Figure S2 The FastQC quality control reports of two biological replicates of wt and BAG-1KO MCF-7 cells. A) The FastQC report of wt MCF-7 cells (replicate 1). B) The FastQC report of wt MCF-7 cells (replicate 2). C) The FastQC report of BAG-1KO MCF-7 cells (replicate 1). D) The FastQC report of BAG-1KO MCF-7 cells (replicate 2).

Figure S3 The FastQC quality control reports of trimmed reads using Cutadapt of wt and BAG-1KO samples. A) The FastQC report of wt MCF-7 cells (replicate 1). B) The FastQC report of wt MCF-7 cells (replicate 2). C) The FastQC report of BAG-1KO MCF-7 cells (replicate 1). D) The FastQC report of BAG-1KO MCF-7 cells (replicate 2).

Figure S4 Sequence count for all annotated miRNAs in the hg19 of the two biological replicates of wt and BAG-1KO MCF-7 using featureCounts.

Figure S5 The featureCount results of 144 miRNAs found in either two replicated wt or BAG-1KO samples with at least 1 TPM.

Figure S6 The featureCount results of 88 miRNAs found in only two replicated wt samples with at least 1 TPM.

Figure S7 The featureCount results of 71 miRNAs found in only two replicated BAG-1KO samples with at least 1 TPM.

Figure S8 The ROSALIND by OnRamp Bio results of differentially expressed miRNA in wt and BAG-1KO MCF-7 cells. A) The log2 counts of reads. B) The percent of total reads.

Figure S9 Differential expression results of 2576 known miRNA counts in BAG-1KO samples against wt samples obtained from the DeSeq2 tool.

Figure S10 The GO functional enrichment analysis includes biological process (A), molecular function (B), and cellular components (C) of 14 downregulated miRNAs with p-values and gene numbers.

Figure S11 The GO functional enrichment analysis including biological process (A), molecular function (B), and cellular components (C) of 11 upregulated miRNAs with p-values and gene numbers.

## Figures and Tables

**Table 1:** Primer sequences for selected miRNA targets for qRT-PCR experiments.

miRNA ID	Forward Primer	Reverse Primer
RNU6	CTCGCTTCGGCAGCACA	AACGCTTCACGAATTTGCGT
hsa-miR-21-5p	GCTAGCTTATCAGACTGATGTTGAAA	GTGCAGGGTCCGAGGT
hsa-miR-429	GCTAATACTGTCTGGTAAAACCGTAA	GTGCAGGGTCCGAGGT
hsa-miR-200a-5p	CGGACAGTGCTGGAAAAA	GTGCAGGGTCCGAGGT
hsa-miR-27a-3p	CAGTGGCTAAGTTCCGCAAA	GTGCAGGGTCCGAGGT
hsa-miR-342-3p	CACAGAAATCGCACCCGTAA	GTGCAGGGTCCGAGGT
hsa-miR-345-5p	GACTCCTAGTCCAGGGCTCAAA	GTGCAGGGTCCGAGGT
hsa-miR-151a-3p	GACTGAAGCTCCTTGAGGAAAA	GTGCAGGGTCCGAGGT
hsa-miR-146b-5p	TGAGAACTGAATTCCATAGGCTAA	GTGCAGGGTCCGAGGT
hsa-miR-31-5p	CAGGCAAGATGCTGGCATAG	GTGCAGGGTCCGAGGT
hsa-miR-887-3p	GCCATCCCGAGGAAAAA	GTGCAGGGTCCGAGGT
hsa-miR-767-5p	CACCATGGTTGTCTGAGCAT	GTGCAGGGTCCGAGGT
hsa-miR-4521	CTAAGGAAGTCCTGTGCTCAGAAA	GTGCAGGGTCCGAGGT
hsa-miR-130b-3p	TGCAATGATGAAAGGGCATA	GTGCAGGGTCCGAGGT
hsa-miR-10a-5p	ACCCTGTAGATCCGAATTTGTG	GTGCAGGGTCCGAGGT

**Table 2:** Significantly differentially expressed 25 miRNAs in BAG-1KO MCF-7 samples comparing wt MCF-7 samples.

Downregulated miRNAs			Upregulated miRNAs		
miRNA ID	Log_2_ fold change	p-value	miRNA ID	Log_2_ fold change	p-value
hsa-miR-21-3p	−6.767	4,97E-11	hsa-miR-92a-3p	1.895	0.013
hsa-miR-21-5p	−6.447	6,37E-248	hsa-miR-30a-3p	2.559	0.008
hsa-miR-429	−5.805	0.000	hsa-miR-130b-3p	2.709	0.008
hsa-miR-296-3p	−5.466	0.001	hsa-miR-148a-5p	2.99	0.006
hsa-miR-200a-5p	−5.388	0.002	hsa-miR-4521	4.055	0.001
hsa-miR-27a-3p	−4.218	0.001	hsa-miR-873-5p	5.602	0.004
hsa-let-7b-5p	−4.115	1,25E-21	hsa-miR-767-5p	5.771	0.002
hsa-let-7i-5p	−3.838	5,99E-28	hsa-miR-887-3p	5.771	0.002
hsa-miR-342-5p	−3.772	0.000	hsa-miR-31-5p	6.539	0.000
hsa-miR-342-3p	−2.875	0.008	hsa-miR-10a-5p	6.939	6,09E-133
hsa-miR-345-5p	−2.131	0.003	hsa-miR-10b-5p	8.538	1,83E-111
hsa-miR-151a-3p	−1.811	0.006			
hsa-miR-98-5p	−4.938	3,55E-26			
hsa-miR-146b-5p	−1.123	0.010			

**Table 3:** Enriched KEGG pathways, differentially expressed miRNAs and miRNA-associated target genes of 14 downregulated miRNAs.

Dowregulate miRNAs					
KEGG pathway	p-value	gene number	target genes	miRNA number	miRNA ID
ECM-receptor interaction	0,00	26	ITGB8, LAMB1, SV2B, THBS1, ITGA8, COL24A1, COL27A1, ITGB6, AGRN, ITGA5, ITGA1, COL3A1, SV2A, RELN, COL1A1, ITGA2, COL11A2, COL1A2, ITGA7, THBS3, COL4A6, SV2C, FN1, TNR, COL5A2, COL4A1	5	hsa-miR-27a-3p hsa-let-7b-5p hsa-let-7i-5p hsa-miR-342-3p hsa-miR-98-5p
Mucin type O-Glycan biosynthesis	4,8E-05	11	GALNT7, GALNTL6, GCNT4, GALNT1, GALNT3, GALNT14, GALNT2, C1GALT1, GALNT12, GALNT5, GALNT16	6	hsa-miR-27a-3p hsa-let-7b-5p hsa-let-7i-5p hsa-miR-342-5p hsa-miR-345-5p hsa-miR-98-5p
Signaling Pathways regulating pluripotency of stem cells	2,1E+01	44	BMI1, FZD7, GSK3B, DVL3, WNT16, PAX6, SMAD2, NRAS, SMAD9, APC, HOXB1, ACVR1B, MAPK14, HAND1, SMARCAD1, INHBA, IGF1R, ZFHX3, KRAS, FZD3, ACVR2B, FZD4, RIF1, SMAD4, LIFR, SKIL, SMAD5, ACVR2A, ACVR1C, IGF1, AKT3, BMPR1A, PIK3CA, IL6ST, ISL1, ID3, DUSP9, SOX2, PCGF3, GRB2, DVL2, KLF4, WNT9A, BMPR2	5	hsa-miR-429 hsa-miR-27a-3p hsa-let-7b-5p hsa-let-7i-5p hsa-miR-98-5p
Metabolism of xenobiotics by cytochrome P450	0,00	2	GSTM3, GSTM2	1	hsa-miR-342-5p
Glycosphingolipid biosynthesis lacto and neolacto series	0,00	4	FUT4, B3GNT1, FUT9, B3GNT2	2	hsa-miR-429 hsa-miR-200a-5p
Proteoglycans in cancer	0,00	57	FZD7, CAMK2D, BRAF, ACTB, HBEGF, WNT16, MET, SMAD2, CBL, NRAS, THBS1, PTCH1, PPP1CC, MAPK14, PXN, ROCK2, FRS2, RDX, ITGA5, IGF1R, EGFR, KRAS, FZD3, RRAS2, MSN, RPS6KB2, COL21A1, VAV2, PTK2, CBLB, ANK3, FZD4, PPP1R12A, PLCG1, CASP3, FLNA, ITGA2, SOS1, PTPN11, PRKX, RAC1, PRKCB, FAS, IGF1, GAB1, AKT3, PIK3CA, SDC2, FN1, CDKN1A, PDPK1, VEGFA, KDR, GRB2, WNT9A, ERBB4, RPS6KB1	3	hsa-miR-429 hsa-miR-27a-3p hsa-miR-98-5p
Glycosaminoglycan biosynthesis chondroitin sulfate dermatan sulfate	0,00	4	UST, CHST3, CHSY3, XYLT2	3	hsa-let-7b-5p hsa-let-7i-5p hsa-miR-98-5p
Amoebiasis	0,01	11	ARG2, COL27A1, COL3A1, CASP3, COL1A, COL1A2, COL4A6, IL10, COL5A, PLCB4, COL4A1	3	hsa-let-7b-5p hsa-let-7i-5p hsa-miR-98-5p
ErbB signaling pathway	0,01	30	CAMK2D, BRAF, GSK3B, HBEGF, CBL, CRKL, PAK2, MAP2K7, PAK7, CDKN1B, EGFR, KRAS, PTK2, CBLB, PLCG1, JUN, SOS1, PAK6, NRG1, PRKCB, GAB1, SHC4, AKT3, PIK3CA, MAP2K4, ABL2, GRB2, ERBB4, MAPK10, RPS6KB1	2	hsa-miR-429 hsa-miR-27a-3p
Lysine degradation	0,02	15	CAMKMT, WHSC1L1, SETD7, ALDH3A2, SETD2, NSD1, ASH1L, COLGALT2, SUV420H1, DOT1L, WHSC1, SUV420H2, EHHADH, KMT2E, KMT2C	3	hsa-miR-429 hsa-miR-27a-3p hsa-miR-345-5p
Glycosaminoglycan biosynthesis keratan sulfate	0,03	6	ST3GAL1, B4GALT1, B3GNT1, ST3GAL2, CHST2, B3GNT2	2	hsa-miR-21-3p hsa-miR-429
PI3K-Akt signaling pathway	0,04	95	PHLPP2, PRLR, GSK3B, TSC1, PDGFRA, GNG13, PPP2R5E, MET, MYB, ITGB8, LAMB1, FGF14, NRAS, PRKAA2, THBS1, PPP2R2C, ITGA8, CREB5, SYK, COL24A1, YWHAG, CDK2, CCND2, COL27A1, ITGB6, IL7, ITGA5, CDKN1B, YWHAB, MTCP1, EFNA3, IGF1R, EGFR, ITGA1, KRAS, CDK6, COL3A1, LPAR6, YWHAQ, RPS6KB2, GHR, CREB1, IKBKB, FGF11, PTK2, BRCA1, THEM4, CCNE2, RELN, COL1A1, FLT1, VEGFC, COL4A3, ITGA2, SOS1, FGF9, KITLG, COL11A2, IRS1, RAC1, INSR, CHRM2, FGF18, NGF, COL1A2, LAMC1, IGF1, ITGA7, BCL2L1, AKT3, EIF4E2, CREB3L2, PIK3CA, COL4A6, FN1, PKN2, CDKN1A, TNR, PDPK1, VEGFA, OSMR, PPP2R1B, KDR, CSF1, GNG5, GRB2, COL5A2, GNB4, NGFR, BCL2L11, RPS6KB1, COL4A1, CHRM1, IL6R, EFNA1	4	hsa-miR-429 hsa-miR-27a-3p hsa-let-7b-5p hsa-miR-98-5p
TGF-β signaling pathway	0,04	20	SMAD2, SMAD9, SMAD3, INHBA, ACVR2B, SMAD4, SMAD5, ACVR2A, GDF6, SP1, ACVR1C, EP300, BMPR1A, IFNG, LTBP1, ID3, NOG, PPP2R1B, BMPR2, RPS6KB1	3	hsa-miR-21-3p hsa-miR-429 hsa-miR-27a-3p

**Table 4:** Enriched KEGG pathways, differentially expressed miRNAs, and miRNA-associated target genes of 11 upregulated miRNAs.

Upregulated miRNAs					
KEGG pathway	p-value	gene number	target genes	miRNA number	miRNA ID
Prion diseases	<1e-325	1	PRNP	2	hsa-miR-30a-3p hsa-miR-130b-3p
Mucin type O-Glycan biosynthesis	<1e-325	7	POC1B-GALNT4, GALNTL6, GALNT13, GALNT4, GALNT8, GALNT1, ST6GALNAC1	3	hsa-miR-873-5p hsa-miR-10a-5p hsa-miR-10b-5p
ECM-receptor interaction	<1e-325	18	SDC1, ITGA8, COL4A5, COL24A1, COL27A1, ITGA5, COL3A1, ITGAV, COL2A1, COL5A1, COL4A4, COL1A2, COL11A1, COL6A3, ITGA6, COL5A3, COL4A1, ITGB3	4	hsa-miR-92a-3p hsa-miR-767-5p hsa-miR-10a-5p hsa-miR-10b-5p
Metabolism of xenobiotics by cytochrome P450	3,4E+00	6	GSTM5, MGST1, GSTM4, ADH4, GSTK1, CYP1B1	2	hsa-miR-130b-3p hsa-miR-31-5p
Morphine addiction	0.01	7	ADRBK2, GABRG2, PDE10A, GNAI3, GNB1, PDE4D, KCNJ6	1	hsa-miR-148b-5p
TGF-beta signaling pathway	0.03	16	FST, SMAD2, INHBB, SMURF2, SMAD3, INHBA, ID4, ACVR1, SKP1, MYC, ZFYVE9, SMAD5, ACVR1C, TGFB2, TGFBR2	2	hsa-miR-130b-3p hsa-miR-148b-5p

**Table 5:** The predicted target genes and target scores of hsa-miR-429 obtained from DIANA-miRPath v.3.0 and miRDB.

Target Score	Gene Symbol	Gene Description
98	RPS6KB1	ribosomal protein S6 kinase B1
98	MSN	moesin
97	CRKL	CRK like proto-oncogene, adaptor protein
97	PPP2R5E	protein phosphatase 2 regulatory subunit B’epsilon
96	PIK3CA	phosphatidylinositol-4,5-bisphosphate 3-kinase
95	CBL	Cbl proto-oncogene
95	WNT16	Wnt family member 16
95	KDR	kinase insert domain receptor
94	FN1	fibronectin 1
94	JUN	Jun proto-oncogene, AP-1 transcription factor subunit
93	B3GNT2	UDP-GlcNAc:betaGal beta-1,3 acetylglucosaminyltransferase 2
93	VEGFA	vascular endothelial growth factor A
91	YWHAG	tyrosine 3-monooxygenase/tryptophan 5-monooxygenase activation protein gamma
91	CHRM2	cholinergic receptor muscarinic 2
90	ITGA1	integrin subunit alpha 1
89	NOG	noggin
87	ROCK2	Rho associated coiled-coil containing protein kinase 2
85	KLF4	Kruppel like factor 4
84	PPP2R1B	protein phosphatase 2 scaffold subunit Abeta
83	ANK3	ankyrin 3
83	SRY-box	2 SRY-box 2
83	SDC2	syndecan 2
83	RDX	radixin
82	EIF4E2	eukaryotic translation initiation factor 4E family member 2
81	MYB	MYB proto-oncogene, transcription factor
81	CCNE2	cyclin E2
80	ACVR1C	activin A receptor type 1C
80	NRG1	neuregulin 1
79	LAMC1	laminin subunit gamma 1
77	IKBKB	inhibitor of nuclear factor kappa B kinase subunit beta
77	EFNA1	ephrin A1
76	ASH1L	ASH1 like histone lysine methyltransferase
76	GAB1	GRB2 associated binding protein 1
76	PAK6	p21 (RAC1) activated kinase 6
76	KMT2C	lysine methyltransferase 2C
74	FUT9	fucosyltransferase 9
74	ACVR2B	activin A receptor type 2B
74	RRAS2	RAS related 2
73	EP300	E1A binding protein p300
69	RAC1	Rac family small GTPase 1
69	FUT4	fucosyltransferase 4
69	CHST2	carbohydrate sulfotransferase 2
67	SHC4	SHC adaptor protein 4
66	PPP2R2C	protein phosphatase 2 regulatory subunit Bgamma
64	PLCG1	phospholipase C gamma 1
64	RELN	reelin
63	PAX6	paired box 6
62	MTCP1	mature T cell proliferation 1
61	RIF1	replication timing regulatory factor 1
61	SMAD5	SMAD family member 5
60	FRS2	fibroblast growth factor receptor substrate 2
56	CREB5	cAMP responsive element binding protein 5
55	COL4A3	collagen type IV alpha 3 chain
54	SMARCAD1	SWI/SNF-related, matrix-associated actin-dependent regulator of chromatin, subfamily a, containing DEAD/H box 1
54	PDPK1	3-phosphoinositide dependent protein kinase 1
50	PRKAA2	protein kinase AMP-activated catalytic subunit alpha 2
50	FGF9	fibroblast growth factor 9

**Table 6:** The top 10 proteins evaluated in the PPI network using “degree parameter.

Gene name	Gene description	Degree
PIK3CA	Phosphatidylinositol 4,5-bisphosphate 3-kinase 110 kDa catalytic subunit alpha	17
KDR	Kinase insert domain receptor (a type III receptor tyrosine kinase	13
GAB1	Growth factor receptor bound protein 2-associated protein 1	12
RAC1	Ras-related C3 botulinum toxin substrate 1	12
VEGFA	Vascular endothelial growth factor A	12
CBL	Cbl proto-oncogene, E3 ubiquitin protein ligase	11
SDC2	Heparan sulfate proteoglycan core protein	10
PLCG1	1-phosphatidylinositol 4,5-bisphosphate phosphodiesterase gamma-1	10
EP300	Protein propionyltransferase p300	9
CRKL	V-crk avian sarcoma virus CT10 oncogene homolog-like	9

**Table 7 t7-turkjbiol-46-2-118:** GO Functional Annotation analysis of the top ten hub genes included essential proteins of predicted target genes for hsa-miR-429.

GO Funtional Annotation				
Biological Process	Description	p-value	Count	Genes
GO:0010628	positive regulation of gene expression	5.0E-7	8	KLF4, ROCK2, FGF9, FN1, MSN, PAX6, RDX, VEGFA
GO:0048015	phosphatidylinositol-mediated signaling	4.4E-8	7	PDPK1, GAB1, FGF9, FRS2, NRG1, PIK3CA, RPS6KB1
GO:0001525	angiogenesis	3.5E-6	7	JUN, EFNA1, FGF9, FN, KDR, PIK3CA, VEGFA
GO:0016477	cell migration	1.7E-5	6	PDPK1, EFNA1, LAMC1, PLCG1, PLCG, SDC2
GO:0007155	cell adhesion	5.6E-2	4	FN1, LAMC1, RAC1, RELN
**Cellular Compartment**	**Description**	**p-value**	**Count**	**Genes**
GO:0005886	plasma membrane	6.2E-4	17	PDPK1, CBL, ROCK2, SHC4, CHRM2, EFNA1, FRS2, ITGA1, KDR, MSN, PIK3CA, PLCG1, RDX, RAC1, RELN, RRAS2, SDC2
GO:0005737	cytoplasm	2.2E-2	16	PDPK1, ASH1L, CBL, EP300, KLF4, FGF9, FRS2, MSN, NRG1, PAX6, PLCG1, RDX, RAC1, RELN, RPS6KB1, VEGFA
GO:0005622	intracellular	7.3E-3	8	PDPK1, ROCK2, SHC4, EFNA1, PIK3CA, PLCG1, RAC1, RRAS2
GO:0016020	membrane	8.3E-2	8	PDPK1, EFNA1, FRS2, ITGA1, NRG1, RAC1, RRAS2, VEGFA
GO:0005925	focal adhesion	5.5E-4	6	PDPK1, ITGA1, MSN, RDX, RAC1, RRAS2
**Molecular Function**	**Description**	**p-value**	**Count**	**Genes**
GO:0005515	protein binding	6.0E-4	27	PDPK1, CRKL, CBL, EP300, GAB1, JUN, KLF4, MYB, ROCK2, SHC4, CREB5, EFNA1, FRS2, FN1, ITGA1, KDR, KMT2C, MSN, PAX6, PIK3CA, PLCG1, RDX, RAC1, RRAS2, RPS6KB1, SDC2, VEGFA
GO:0046934	phosphatidylinositol-4,5-bisphosphate 3-kinase activity	3.9E-6	5	GAB1, FGF9, FRS2, NRG1, PIK3CA
GO:0019901	protein kinase binding	5.3E-3	5	PDPK1, MSN, PAX6, PLCG1, RAC1
GO:0030971	receptor tyrosine kinase binding	7.3E-5	4	EFNA1, ITGA1, MSN, NRG1
GO:0005178	integrin binding	1.7E-2	3	FN1, KDR, NRG1

**Table 8:** KEGG Pathway enrichment analysis of the top ten essential gene targets for hsa-miR-429.

KEGG Pathway Enrichment			
Description	p-value	Count	Genes
Proteoglycans in cancer	2.0E-15	16	PDPK1, CBL, GAB1, ROCK2, FRS2, FN1, KDR, MSN, PIK3CA, PLCG1, RDX, RAC1, RRAS2, RPS6KB, SDC2, VEGFA
PI3K-Akt signaling pathway	1.0E-10	15	PDPK1, MYB, CREB5, CHRM2, EFNA1, FGF9, FN1, ITGA1, KDR, LAMC1, PIK3CA, RAC1, RELN, RPS6KB1, VEGFA
Focal adhesion	5.0E-11	13	PDPK1, CRKL, JUN, ROCK2, SHC4, FN1, ITGA1, KDR, LAMC1, PIK3CA, RAC1, RELN, VEGFA
Pathways in cancer	8.0E-7	12	CRKL, CBL, EP300, JUN, ROCK2, FGF9, FN1, LAMC1, PIK3CA, PLCG1, RAC1, VEGFA
Regulation of actin cytoskeleton	2.0E-8	11	CRKL, ROCK2, CHRM2, FGF9, FN1, ITGA1, MSN, PIK3CA, RDX, RAC1, RRAS2
Ras signaling pathway	5.5E-7	10	GAB1, SHC4, EFNA1, FGF9, KDR, PIK3CA, PLCG1, RAC1, RRAS2, VEGFA
