# Supplementary figures and images for: The comparison of differentially expressed microRNAs in Bag-1 deficient and wild type MCF-7 breast cancer cells by small RNA sequencing
Source: Turk J Biol. 2021 Nov 14;46(2):118–36. doi: 10.3906/biy-2109-48 (PMC10393109; doi:10.3906/biy-2109-48)

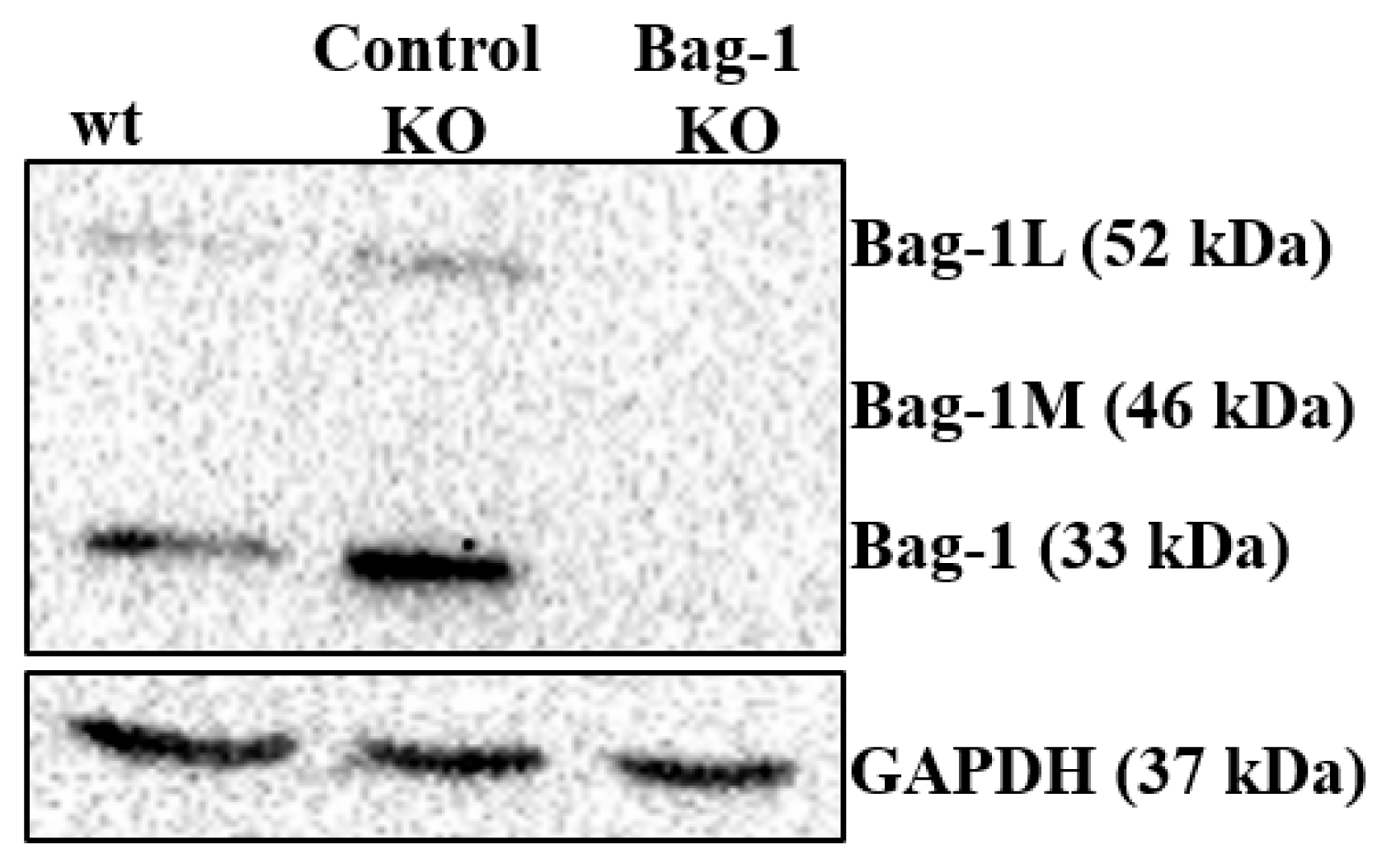

Supplement: Figure S1 — Determination of the loss of Bag-1 expression by immunoblotting. GAPDH was used as a loading control. [file turkjbiol-46-2-118s1.tif]

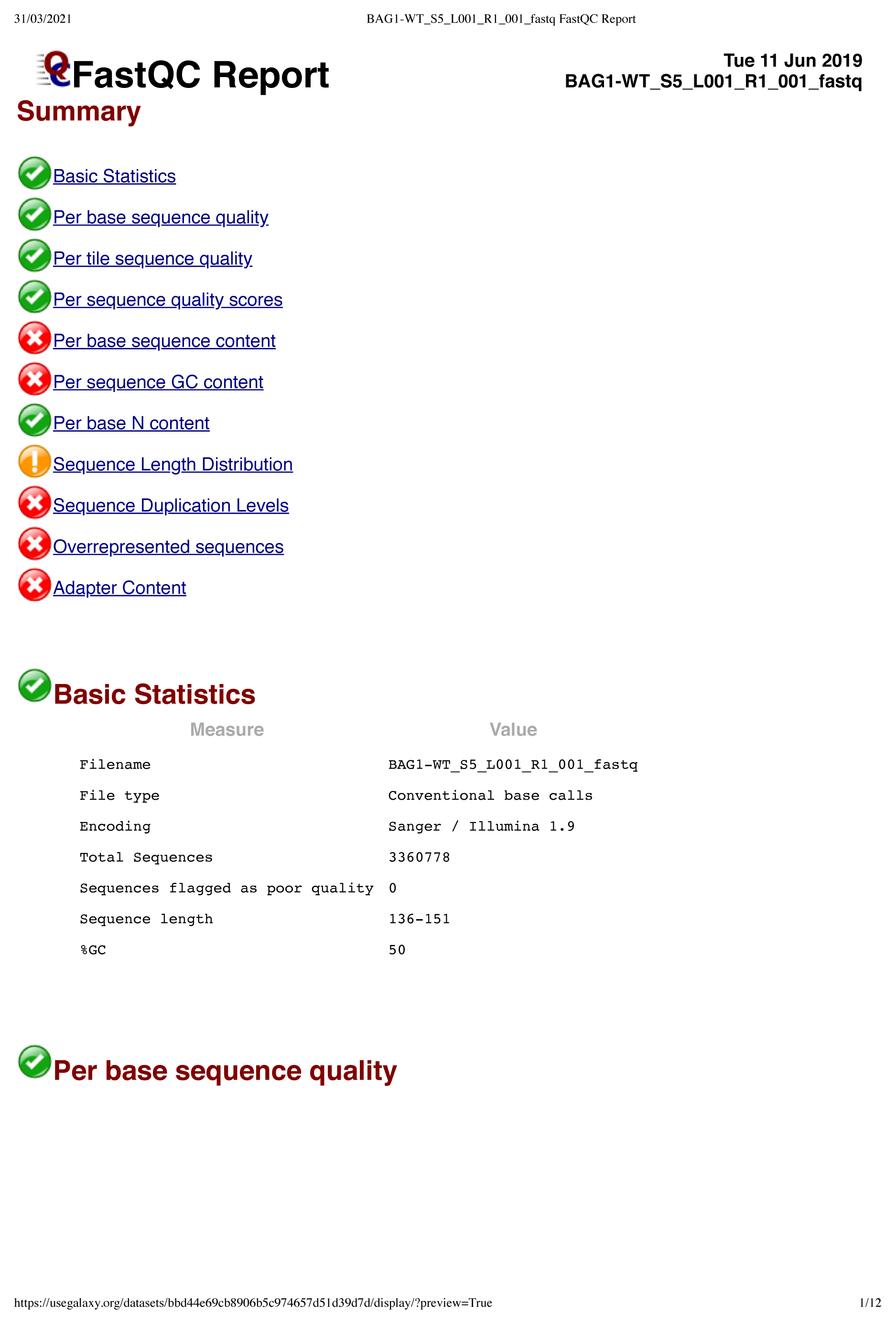

Supplement: Figure S2 — The FastQC quality control reports of two biological replicates of wt and BAG-1KO MCF-7 cells. A) The FastQC report of wt MCF-7 cells (replicate 1). B) The FastQC report of wt MCF-7 cells (replicate 2). C) The FastQC report of BAG-1KO MCF-7 cells (replicate 1). D) The FastQC report of BAG-1KO MCF-7 cells (replicate 2). [file turkjbiol-46-2-118s2a.tif]

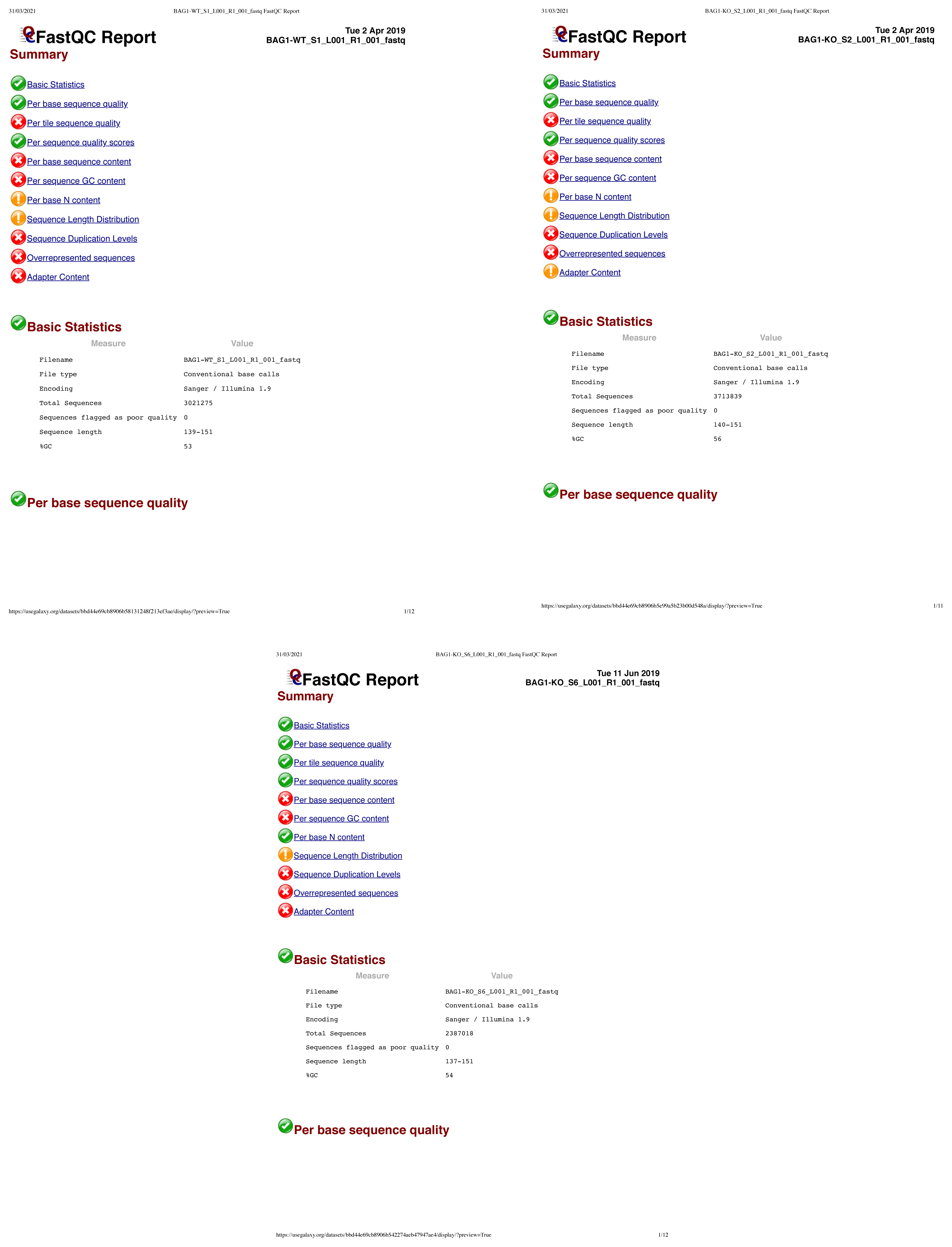

Supplement: Figure S2 — The FastQC quality control reports of two biological replicates of wt and BAG-1KO MCF-7 cells. A) The FastQC report of wt MCF-7 cells (replicate 1). B) The FastQC report of wt MCF-7 cells (replicate 2). C) The FastQC report of BAG-1KO MCF-7 cells (replicate 1). D) The FastQC report of BAG-1KO MCF-7 cells (replicate 2). [file turkjbiol-46-2-118s2b.tif]

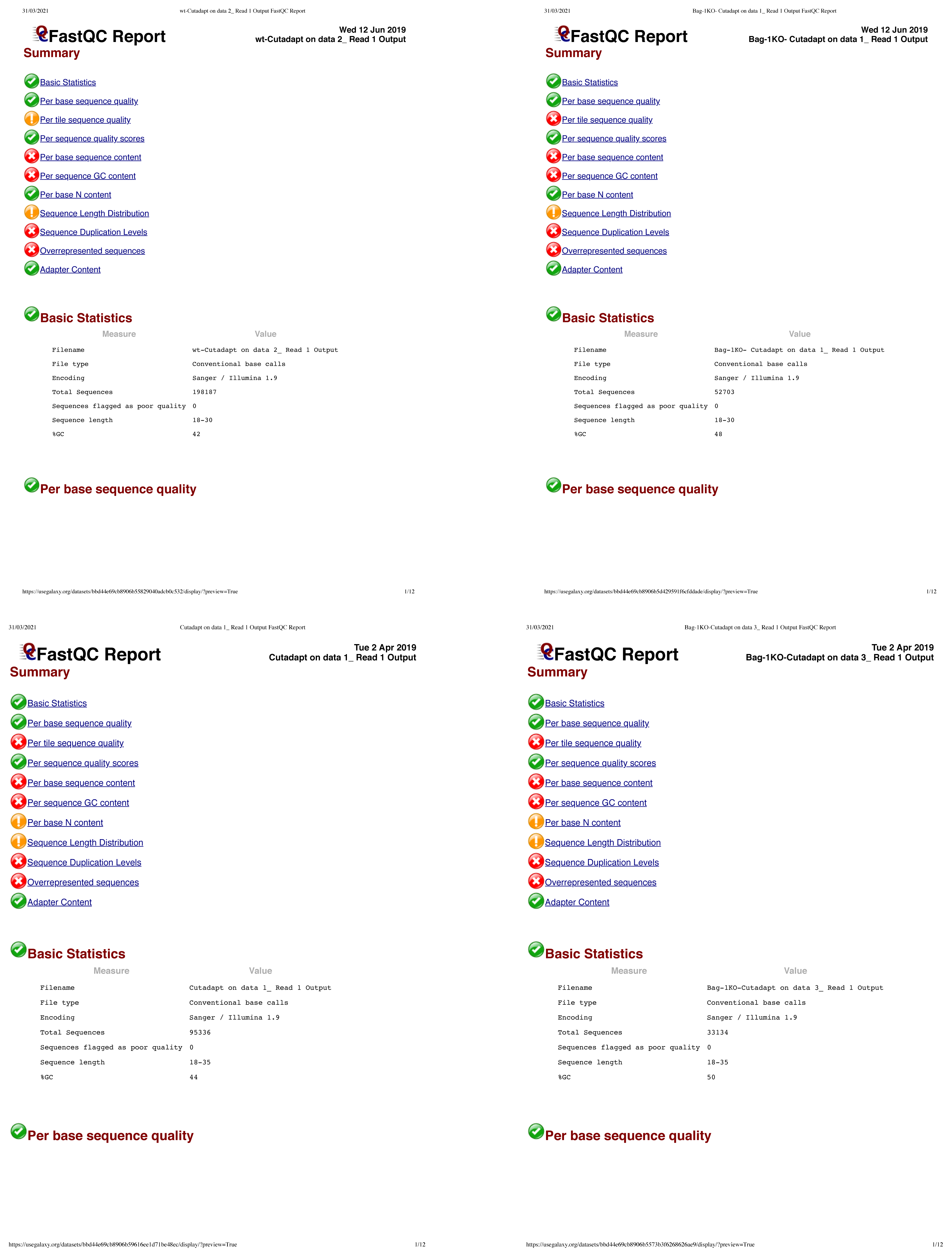

Supplement: Figure S3 — The FastQC quality control reports of trimmed reads using Cutadapt of wt and BAG-1KO samples. A) The FastQC report of wt MCF-7 cells (replicate 1). B) The FastQC report of wt MCF-7 cells (replicate 2). C) The FastQC report of BAG-1KO MCF-7 cells (replicate 1). D) The FastQC report of BAG-1KO MCF-7 cells (replicate 2). [file turkjbiol-46-2-118s3.tif]

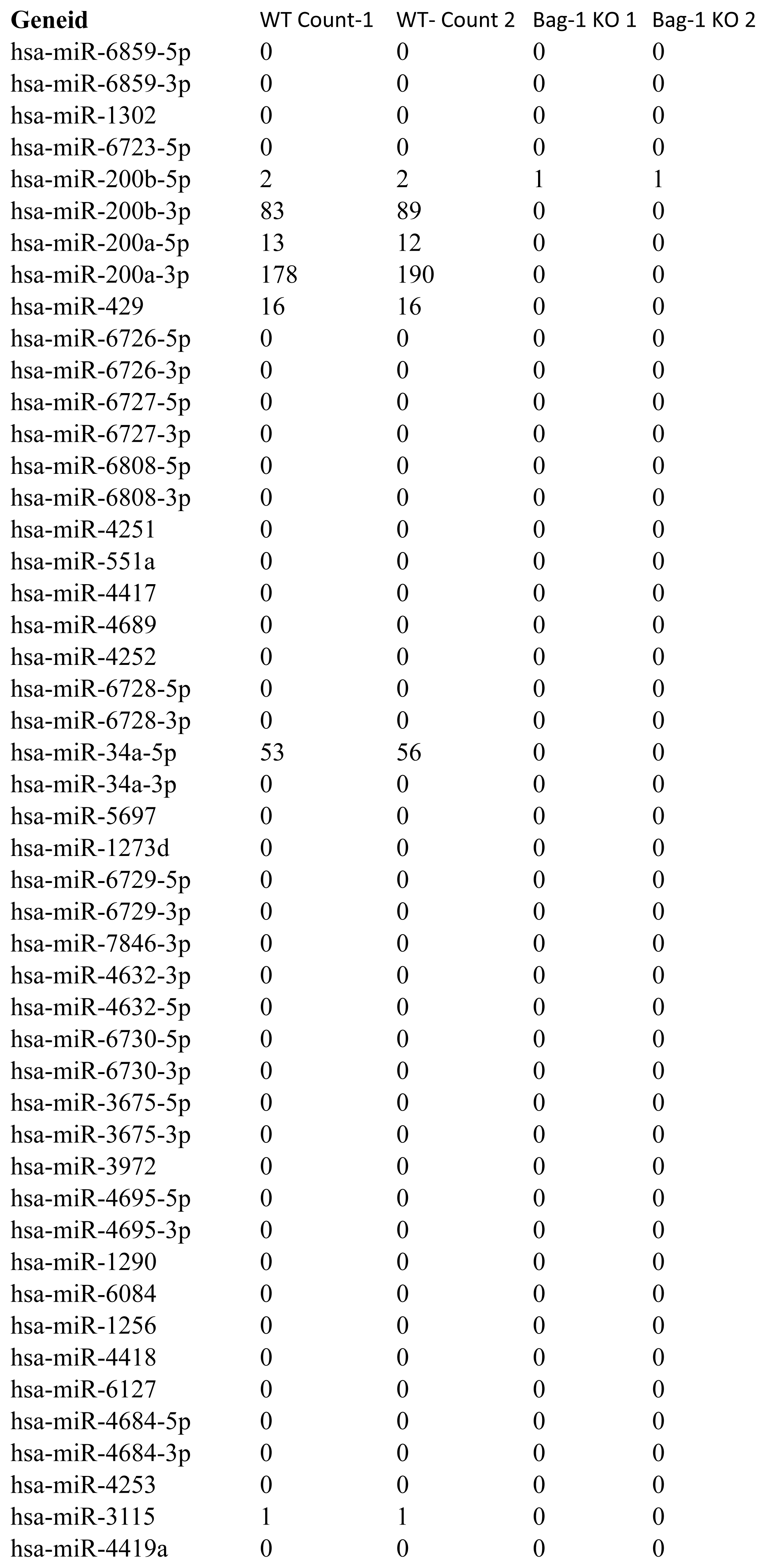

Supplement: Figure S4 — Sequence count for all annotated miRNAs in the hg19 of the two biological replicates of wt and BAG-1KO MCF-7 using featureCounts. [file turkjbiol-46-2-118s4.tif]

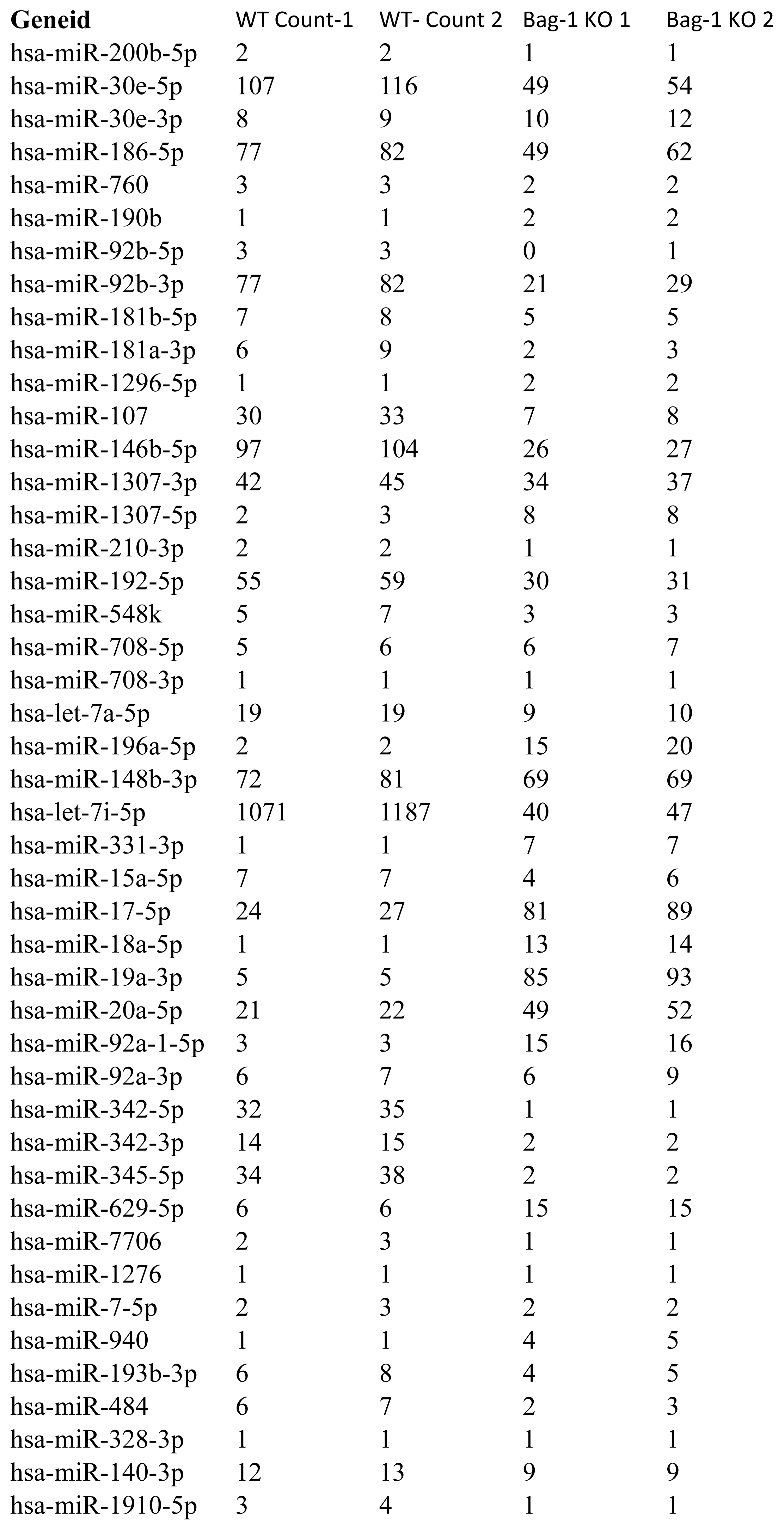

Supplement: Figure S5 — The featureCount results of 144 miRNAs found in either two replicated wt or BAG-1KO samples with at least 1 TPM. [file turkjbiol-46-2-118s5.tif]

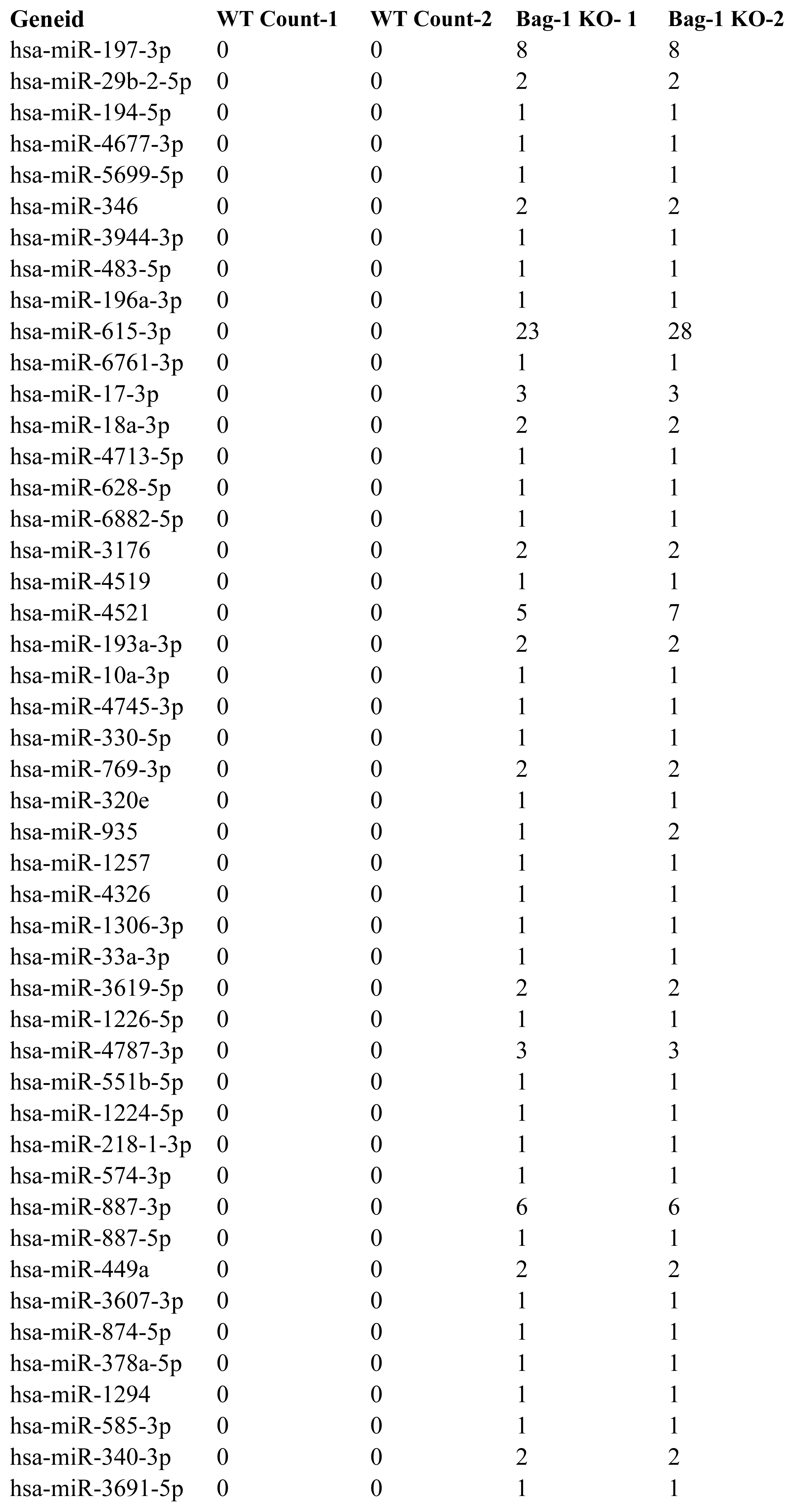

Supplement: Figure S6 — The featureCount results of 88 miRNAs found in only two replicated wt samples with at least 1 TPM. [file turkjbiol-46-2-118s6.tif]

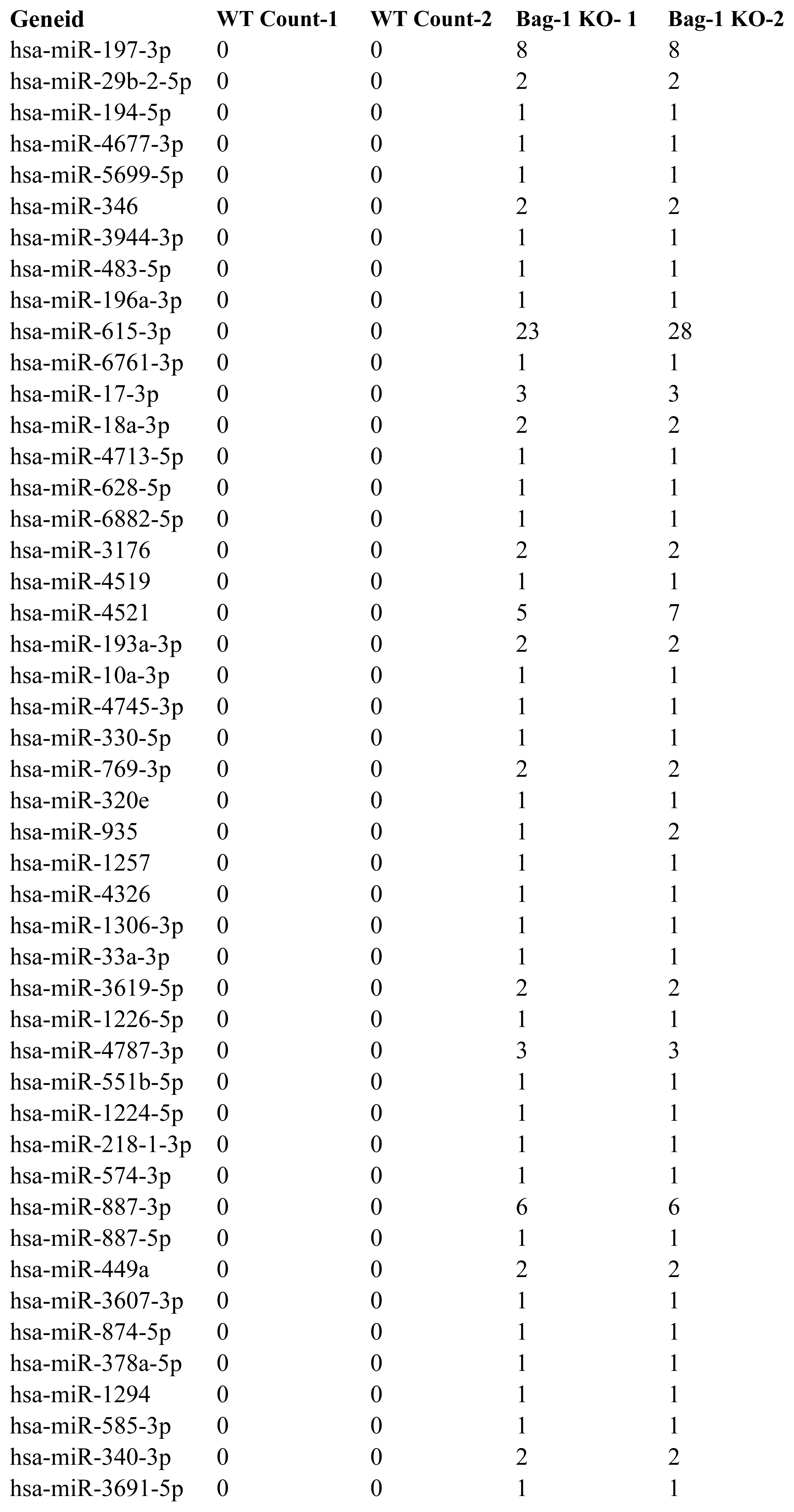

Supplement: Figure S7 — The featureCount results of 71 miRNAs found in only two replicated BAG-1KO samples with at least 1 TPM. [file turkjbiol-46-2-118s7.tif]

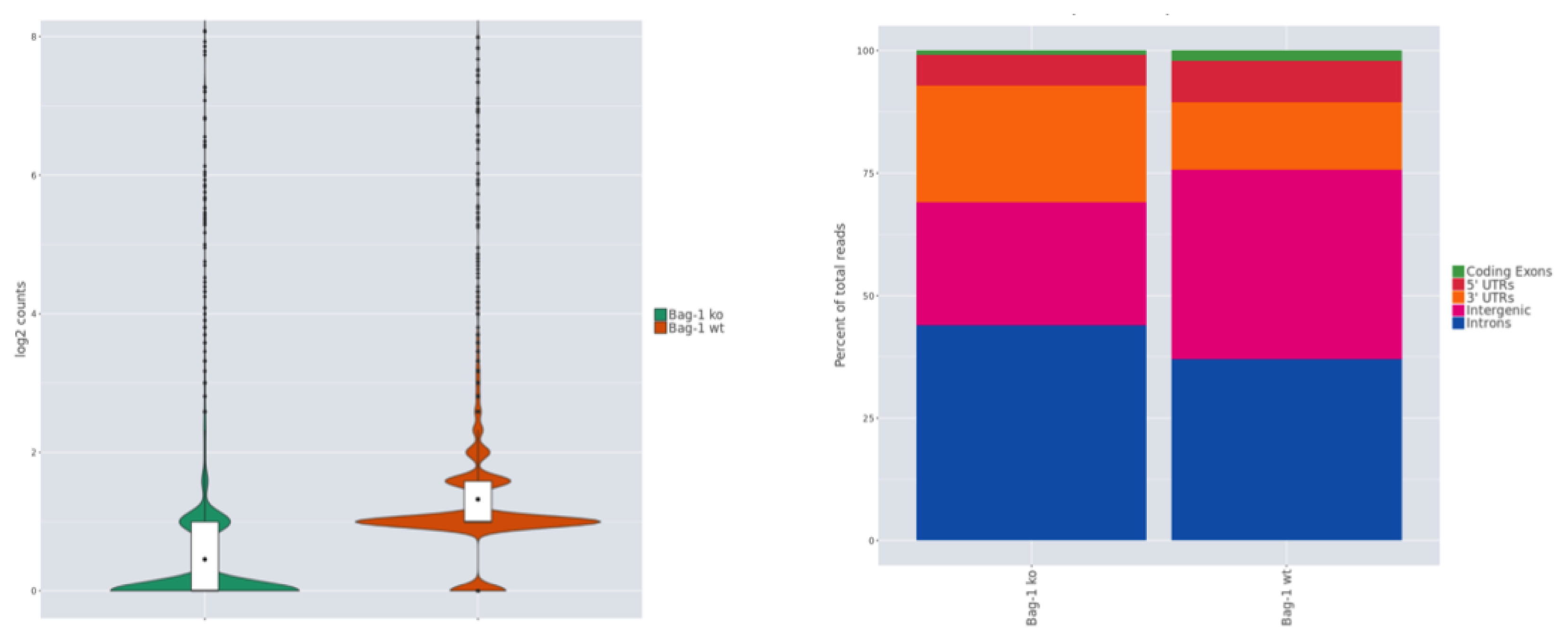

Supplement: Figure S8 — The ROSALIND by OnRamp Bio results of differentially expressed miRNA in wt and BAG-1KO MCF-7 cells. A) The log2 counts of reads. B) The percent of total reads. [file turkjbiol-46-2-118s8.tif]

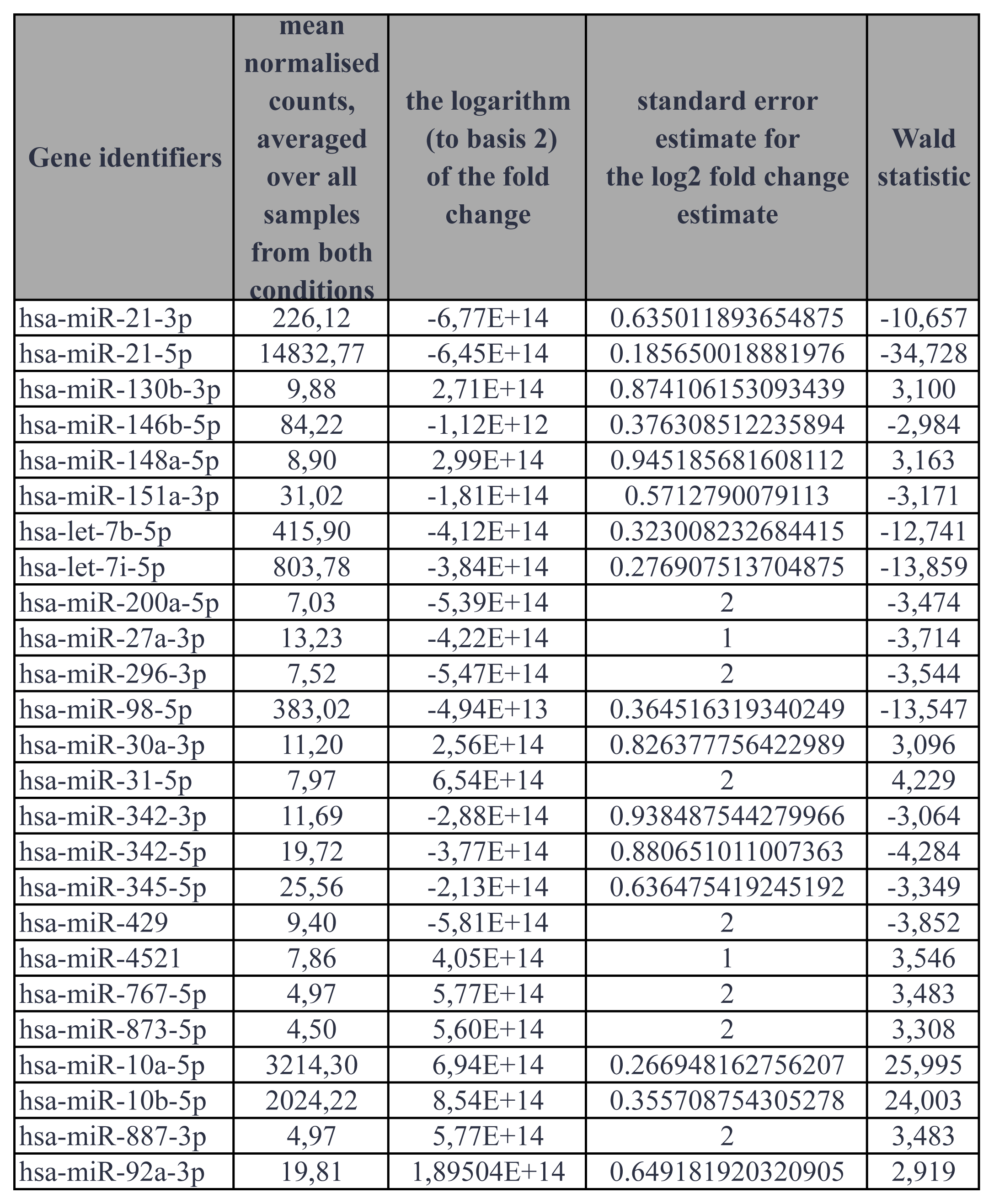

Supplement: Figure S9 — Differential expression results of 2576 known miRNA counts in BAG-1KO samples against wt samples obtained from the DeSeq2 tool. [file turkjbiol-46-2-118s9.tif]

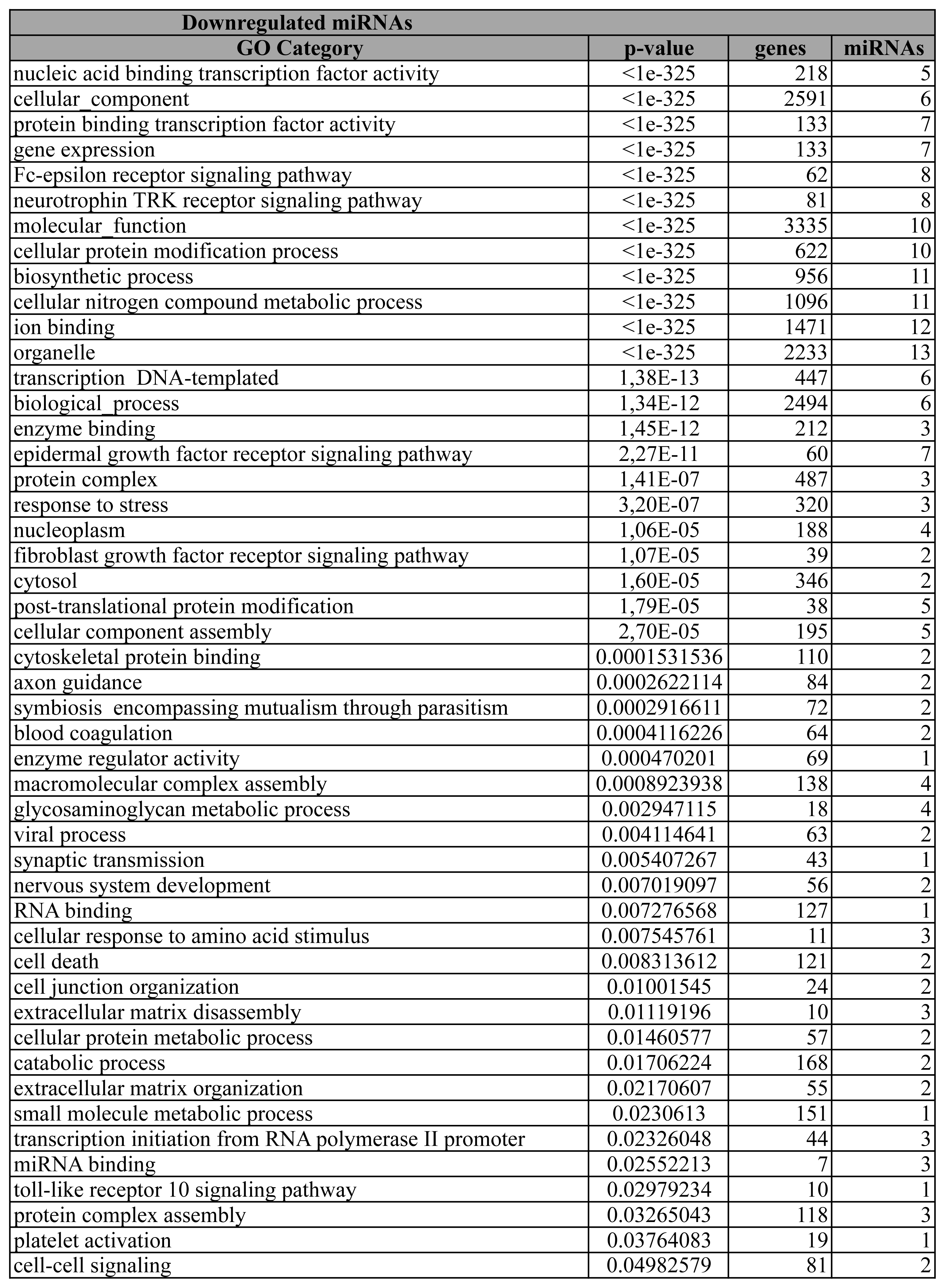

Supplement: Figure S10 — The GO functional enrichment analysis includes biological process (A), molecular function (B), and cellular components (C) of 14 downregulated miRNAs with p-values and gene numbers. [file turkjbiol-46-2-118s10a.tif]

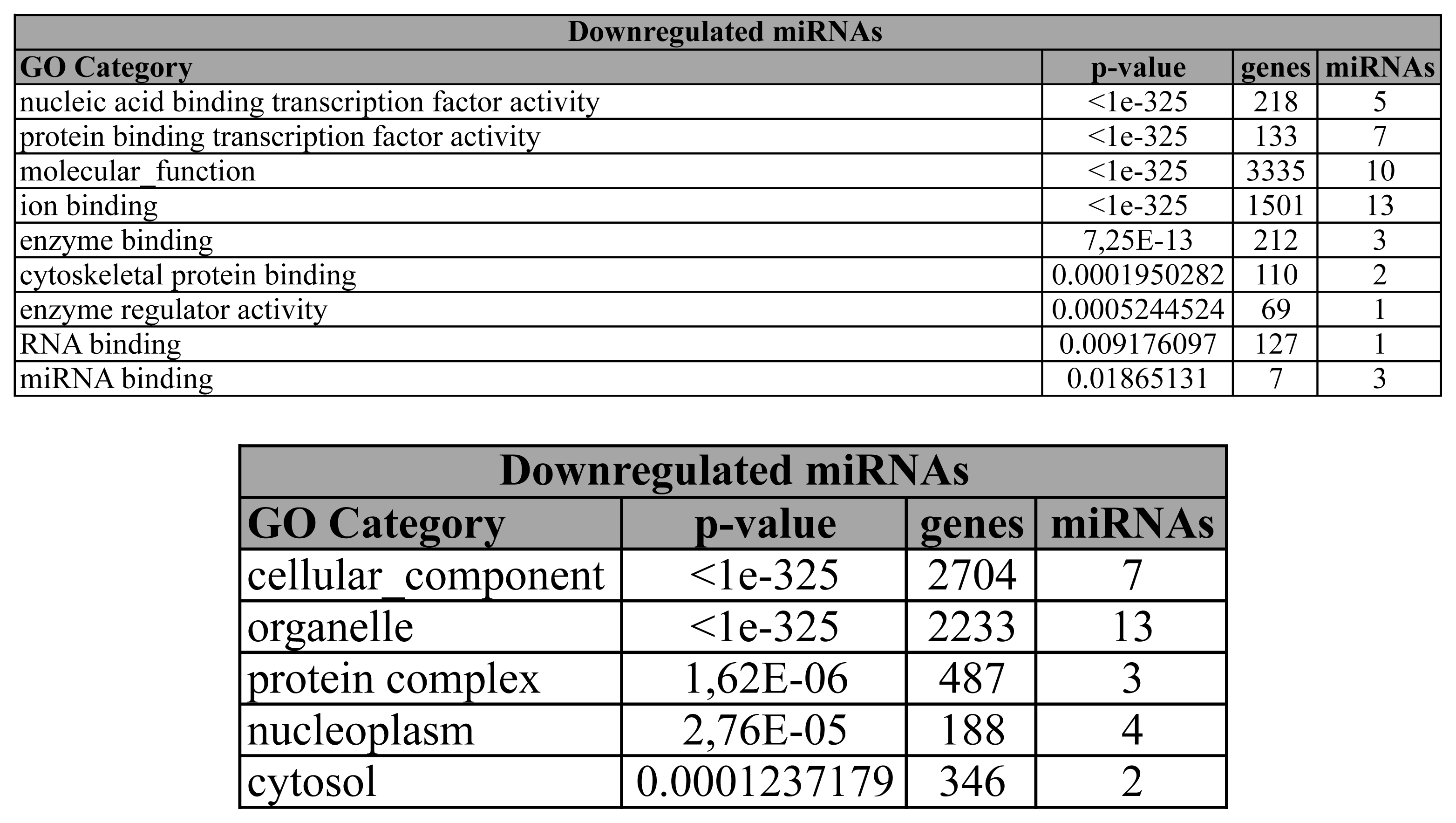

Supplement: Figure S10 — The GO functional enrichment analysis includes biological process (A), molecular function (B), and cellular components (C) of 14 downregulated miRNAs with p-values and gene numbers. [file turkjbiol-46-2-118s10b.tif]

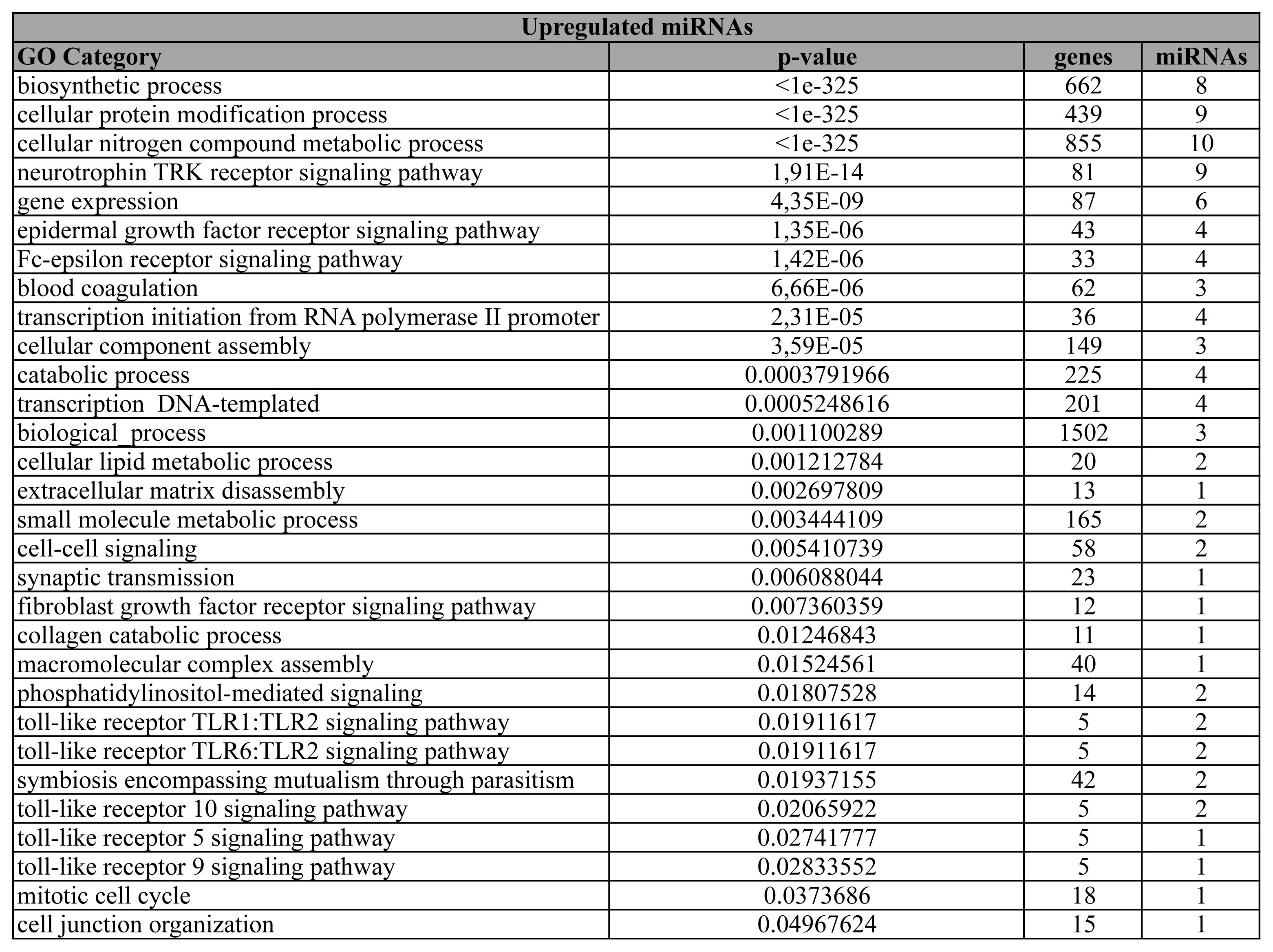

Supplement: Figure S11 — The GO functional enrichment analysis including biological process (A), molecular function (B), and cellular components (C) of 11 upregulated miRNAs with p-values and gene numbers. [file turkjbiol-46-2-118s11a.tif]

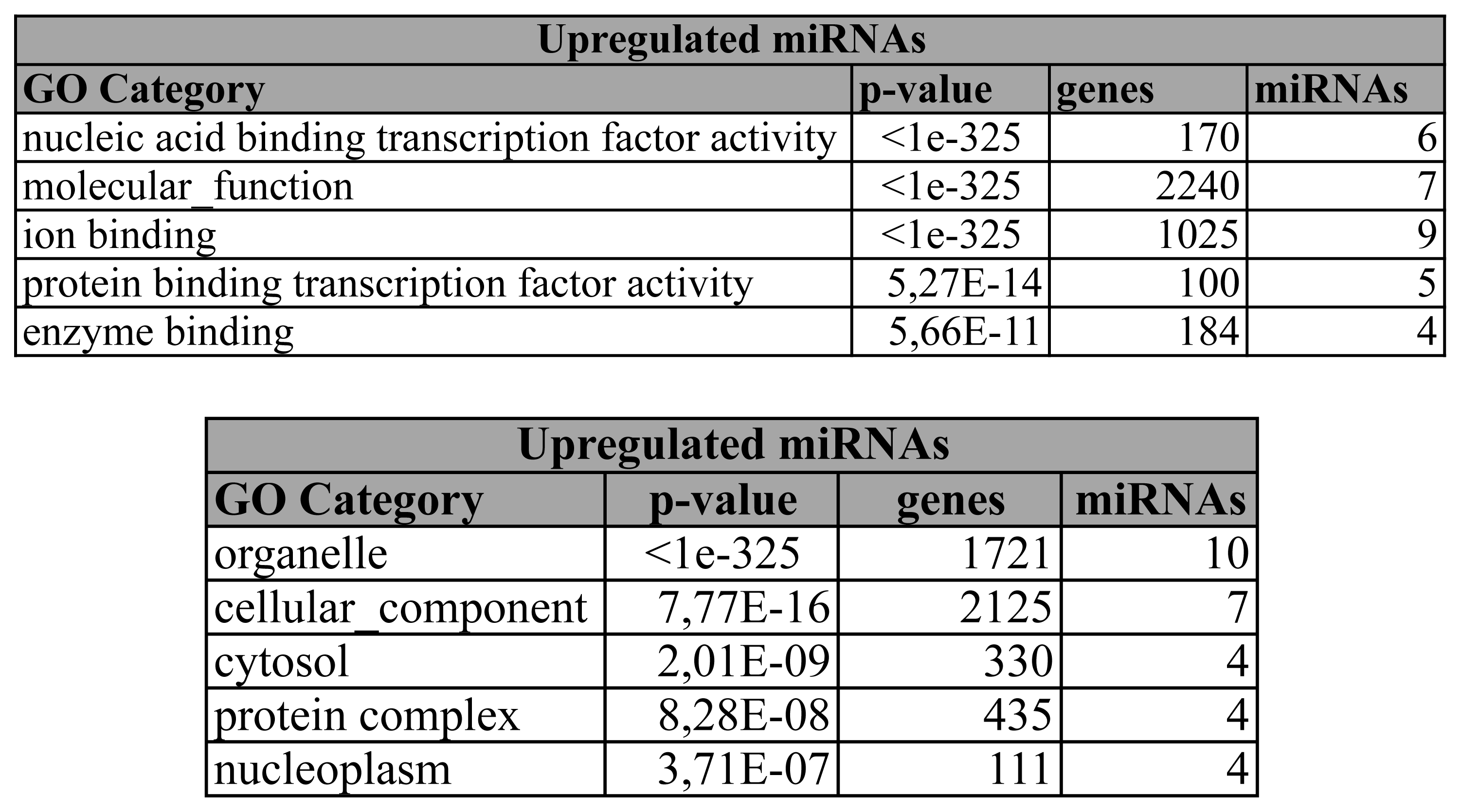

Supplement: Figure S11 — The GO functional enrichment analysis including biological process (A), molecular function (B), and cellular components (C) of 11 upregulated miRNAs with p-values and gene numbers. [file turkjbiol-46-2-118s11b.tif]
